# Converging blockchain and next-generation artificial intelligence technologies to decentralize and accelerate biomedical research and healthcare

**DOI:** 10.18632/oncotarget.22345

**Published:** 2017-11-09

**Authors:** Polina Mamoshina, Lucy Ojomoko, Yury Yanovich, Alex Ostrovski, Alex Botezatu, Pavel Prikhodko, Eugene Izumchenko, Alexander Aliper, Konstantin Romantsov, Alexander Zhebrak, Iraneus Obioma Ogu, Alex Zhavoronkov

**Affiliations:** ^1^ Pharmaceutical Artificial Intelligence Department, Insilico Medicine, Inc., Emerging Technology Centers, Johns Hopkins University at Eastern, Baltimore, Maryland, USA; ^2^ Department of Computer Science, University of Oxford, Oxford, United Kingdom; ^3^ The Bitfury Group, Amsterdam, Netherlands; ^4^ Department of Otolaryngology-Head & Neck Surgery, Johns Hopkins University School of Medicine, Baltimore, MD, USA; ^5^ Africa Blockchain Artificial Intelligence for Healthcare Initiative, Insilico Medicine, Inc, Abuja, Nigeria; ^6^ The Biogerontology Research Foundation, London, United Kingdom

**Keywords:** artificial intelligence, deep learning, data management, blockchain, digital health

## Abstract

The increased availability of data and recent advancements in artificial intelligence present the unprecedented opportunities in healthcare and major challenges for the patients, developers, providers and regulators. The novel deep learning and transfer learning techniques are turning any data about the person into medical data transforming simple facial pictures and videos into powerful sources of data for predictive analytics. Presently, the patients do not have control over the access privileges to their medical records and remain unaware of the true value of the data they have. In this paper, we provide an overview of the next-generation artificial intelligence and blockchain technologies and present innovative solutions that may be used to accelerate the biomedical research and enable patients with new tools to control and profit from their personal data as well with the incentives to undergo constant health monitoring. We introduce new concepts to appraise and evaluate personal records, including the combination-, time- and relationship-value of the data. We also present a roadmap for a blockchain-enabled decentralized personal health data ecosystem to enable novel approaches for drug discovery, biomarker development, and preventative healthcare. A secure and transparent distributed personal data marketplace utilizing blockchain and deep learning technologies may be able to resolve the challenges faced by the regulators and return the control over personal data including medical records back to the individuals.

## INTRODUCTION

The digital revolution in medicine produced a paradigm shift in the healthcare industry. One of the major benefits of the digital healthcare system and electronic medical records is the improved access to the healthcare records both for health professionals and patients. The success of initiatives that provides patients with the access to their electronic healthcare records, such as OpenNotes, suggests their potential to improve the quality and efficiency of medical care [1, 2]. At the same time, biomedical data is not limited to the clinical records created by physicians, the substantial amount of data is retrieved from biomedical imaging, laboratory testing such as basic blood tests, and omics data. Notably, the amount of genomic data alone is projected to surpass the amount of data generated by other data-intensive fields such as social networks and online video-sharing platforms [3]. National healthcare programs such as the UK Biobank (supported by the National Health Service (NHS)) [4] or global programmes such as the LINCS consortium (http://www.lincsproject.org/) and the ENCODE project (https://www.encodeproject.org/), provide scientists with tens of thousands of high-quality samples. However, while increased data volume and complexity offers new exciting perspectives in healthcare industry development, it also introduces new challenges in data analysis and interpretation, and of course, privacy and security. Due to huge demand for the treatments and prevention of chronic diseases, mainly driven by aging of the population, there is a clear need for the new global integrative healthcare approaches [5]. Majority of the recent approaches to personalized medicine in oncology and other diseases relied on the various data types including the multiple types of genomic [6-10], transcriptomic [11-13], microRNA [14], proteomic [15], antigen [16], methylation [17], imaging [18, 19], metagenomic [20], mitochondrial [21], metabolic [22], physiological [23] and other data. And while several attempts were made to evaluate the clinical benefit of the different methods [24] and multiple data types were used for evaluating the health status of the individual patients [25] including the widely popularized “Snyderome” project [26], none of these approaches are truly integrative on the population scale and compare the predictive nature and value of the various data types in the context of biomedicine. Introduction of new technologies, such as an artificial intelligence and blockchain, may enhance and scale up the progress in health care sciences and lead to effective and cost-efficient healthcare ecosystems.

In this article we first review one of the recent achievements in next-generation artificial intelligence, deep learning, that holds the great promise as a biomedical research tool with many applications. We then discuss basic concepts of highly distributed storage systems (HDSS) as one of the advantageous solutions for data storage, introduce the open-source blockchain framework Exonum and review the application of blockchain for healthcare marketplace. For the first time we introduce *half-life period* of analysis significance, models of data value for single and group of users and cost of buying data in the context of biomedical applications. WHere we also present a blockchain-based platform for empowering patients to ensure that they havetake a control over their personal data, manage the access priviledges and to protect their data privacy, as well to allow patients to benefit from their data receiving a crypto tokesscurrency as a reward for their data or for healthy behaviorand to contribute to the overall biomedical progress. We speculate that such systems may be used by the governments on the national scale to increase participation of the general public in preventative medicine and even provide the universal basic income to the their citizens willing to participate in such programs that will greatly decrease the burden of disease on the healthcare systems. Finally, we cover important aspects of data quality control using the recent advances in deep learning and other machine learning methods.

## ADVANCES IN ARTIFICIAL INTELLIGENCE

While the amount of health-associated data and the number of large scales global projects increases, integrative analysis of this data is proving to be problematic [27]. Even high-quality biomedical data is usually highly heterogeneous and complex and requires special approaches for preprocessing and analysis. Computational biology methods are routinely used in various fields of healthcare and are incorporated in pipelines of pharmaceutical companies. Machine learning techniques are among the leading and the most promising tools of computational analysis.

Increased computer processing power and algorithmic advances have led to the significant improvement in the field machine learning. Although machine learning methods are now routinely used in various research fields, including biomarker development and drug discovery [28-31], the machine learning techniques utilizing the Deep Neural Networks (DNNs) are able to capture high-level dependencies in the healthcare data [32]. The feedforward DNNs were recently successfully applied to prediction the various drug properties, such as pharmacological action [33, 34], and toxicity [35]. Biomarker development, a design or search for distinctive characteristics of healthy or pathological conditions, is another area where the application of DNNs has led to significant achievements. For example, an ensemble of neural networks was applied to predict age and sex of patients based on their common blood test profiles [36]. Convolutional neural networks (CNNs) were trained to classify cancer patients using immunohistochemistry of tumour tissues [37]. And in early 2017, first neural network based platform, called Arterys Cardio DL, was officially approved by US Food and Drug Administration (FDA) [38] and is currently used in the clinic.

While the DNNs are able to extract features from the data automatically and usually outperform the other machine learning approaches in feature extraction tasks, one of the good practices is to select a set of relevant features before training the deep model, especially when the dataset is comparatively small. Algorithms such as the principal component analysis or clustering methods are widely used in bioinformatics [39]. However, these first-choice approaches transform the data into a set of components and features that may be difficult hard to interpret from the perspective of biology. Supervised knowledge-based approaches such as pathway or network analysis, on the other hand, provide an attractive alternative, allowing for reduction of a number of input elements and preserving the biological relevance at the same time, which is crucial for the claimed to be hard to interpret “black box” methods such as DNNs. For example, Aliper and colleagues used signaling pathway analysis to reduce the dimensionality of drug-induced gene expression profiles and to train a DNN based predictor of pharmacological properties of drugs [33]. Selected pathway activation scores were compared to expression changes of over 1000 most representative, landmark genes. DNN trained on the pathway scores outperformed DNN trained on the set of landmark genes and achieved the F1 score, the weighted average of precision and recall, of 0.701 for the three drug pharmacological classes. In addition, signaling pathway-based dimensionality reduction allowed for the more robust performance on the validation set, while classifiers trained on gene expression data demonstrated a significant decrease in predictive accuracy on the validation set compared to the training set performance.

There are many promising machine learning techniques in practice and in development including the upcoming capsule networks and recursive cortical networks and many advances are being made in symbolic learning and natural language processing. However, the recurrent neural networks, generative adversarial networks and transfer learning techniques are gaining popularity in the healthcare applications and can be applied to the blockchain-enabled personal data marketplaces.

### Generative adversarial networks

Generative adversarial networks (GANs) are among the most promising recent developments in deep learning. GAN architecture was first introduced by Goodfellow et al. in 2014 [40] and already demonstrated compelling results in image and text generation. Similar concepts were applied for molecule generation by Kadurin and colleagues [41]. A dataset of molecules with the different tumor growth inhibition (TGI) activity was used to train an adversarial Autoencoder (AAE), which combines the properties of both the discriminator and the generator. The trained model then was used to generate the fingerprints of molecules with desired properties. Further analysis of the generated molecules showed that new molecular fingerprints are matched closely to already known highly effective anticancer drugs such as anthracyclines. As a continuation of this work, authors proposed an enhanced architecture that also included additional molecular parameters such as solubility and enabled the generation of more chemically diverse molecules [42]. New model clearly showed the improvement in the training and generation processes, suggesting a great potential in drug discovery.

### Recurrent neural networks

Electronic health records contain the clinical history of patients and could be used to identify the individual risk of developing cardiovascular diseases, diabetes and other chronic conditions [43]. Recurrent neural networks (RNNs), which are naturally suited for sequence analysis, are one of the most promising tools for text or time-series analysis. And one of the most advantageous applications of RNNs in healthcare is electronic medical record analysis. Recently, RNNs were used to predict heart failure of patients based on clinical events in their records [44]. Models trained on 12 month period of clinical history and tested on 6 months demonstrated an Area Under the Curve (AUC) of 0.883 and outperformed shallow models. Interestingly, analysis of cases that were predicted incorrectly, showed that networks tend to predict heart failure based on a patient history of heart diseases, for example hypertension. At the same time, most of the false negative heart failure predictions are made for cases of acute heart failure with little or no symptoms. Along with cardiovascular disease risk prediction, RNNs were also applied to predict blood glucose level of Type I diabetic patients (up to one hour) using data from continuous glucose monitoring devices [45]. The proposed system operates fully automatically and could be integrated with blood glucose and insulin monitoring systems.

While mobile health is an attractive and promising field that emerged recently, another exciting area of RNNs application is human activity prediction based on data from wearable devices. For example, RNN model called DeepConvLSTM, a model that combine convolutional networks and recurrent networks with Long Short-Term Memory (LSTM) architecture was applied on recordings from on-body sensors to predict movements and gestures [46]. Those technologies hold the most potential in distance chronic disease monitoring such as Parkinson’s [47] and cardiovascular diseases [48].

### Transfer learning

Being exceptionally data hungry, most of deep learning algorithms require a lot of data to train and test the system. Many approaches have been proposed to address this problem, including transfer learning. Transfer learning focuses on translating information learned on one domain or larger dataset to another domain, smaller in size. Transfer learning techniques are commonly used in image recognition when the large data sets required to train the deep neural networks to achieve high accuracy are not available. The architecture of CNNs allows transferring fitted parameters of a trained neural network to another network. Biomedical image datasets are usually limited by the size of samples, so larger non-biological image collections, such ImageNet, could be used to fine-tune a network first. A CNN pre-trained on the ImageNet was further trained on magnetic resonance images (MRIs) of heart to outline the organ structure [49]. With an average F1 score of 97.66%, the proposed model achieved state-of-the-art cardiac structure recognition. Similarly, CNNs fined-tuned on the ImageNet were applied for glioblastoma brain tumour prediction [50].

### One and zero-shot learning

One and zero-shot learning are some of the transfer learning techniques that allow to deal with restricted datasets. Taking into account that real-world data is usually imbalanced, one shot learning is aimed to recognise new data points based on only a few examples in the training sets. Going further, zero-shot learning intents to recognise new object without seeing the examples of those instances in the training set. Both one and zero-shot learning are concepts of the transfer learning.

Medical chemistry is one of the fields where data is scarce, therefore, to address this problem Altae-Tran and colleagues proposed a one-shot learning approach for the prediction of molecule toxic potential [51]. In this work, authors use a graph representation of molecules linked to the labels from Tox21 and SIDER databases to train and test models. One-shot networks as siamese networks, LSTMs with attention and novel Iterative Refinement LSTMs, were compared with each other, with graph convolutional neural networks and with random forest with 100 trees as a conventional model. Iterative Refinement LSTMs outperformed other models on most of the Tox21 assays and SIDER side effect. In addition, to evaluate the translational potential of the one-shot architecture, networks trained on Tox21 data were tested on SIDER, however none of the one-shot networks achieved any predictive power, highlighting the potential limitation in translation from toxic *in vitro* assays into the human clinic.

## HIGHLY DISTRIBUTED STORAGE SYSTEMS

The recent explosion in generation and need for data has made it very necessary to find better systems for data storage. Among other requirements, the data storage systems should be better in terms of reliability, accessibility, scalability and affordability, all of which would translate into improved availability. While there could be many options for optimizing these requirements, HDSS has been found to be a very useful and viable option. Traditionally, a lot of technologies and techniques have been employed to store data since the development of computer systems, however, with the exponential increase in data demands and computing power, solutions like HDSS has become very important.

Basically, HDSS involves storing data in multiple nodes, which could simply be databases or host computers. Data stored in these nodes are usually replicated or redundant and HDSS makes a quick access to data over this large number of nodes possible. It is usually specifically used to refer to either a distributed database where users store information on a number of nodes, or a computer network in which users store information on a number of peer network nodes.

In recent years, storage failures have been one of the data handling challenges of higher importance, making reliability one of the important requirements for storage systems. HDSS, which allows data to be replicated in a number of different nodes or storage units and makes it protected from failures, has become very popular.

### Advances in HDSS

There have been a significant amount of progress both in the applications and the optimization of HDSS. However, some of the key challenges in HDSS applications are ensuring consistency of data across various storage nodes and affordability of the systems. These challenges have been addressed by many recent HDSS solutions, including distributed non-relational databases and peer network node data stores. This is for example, a case of peer-to-peer node data store implemented in blockchain.

Blockchain could be described as a distributed database that is used to maintain a continuously growing list of records. These records are composed into blocks, which are locked together using certain cryptographic mechanisms to maintain consistency of the data. Normally a blockchain is maintained by a peer-to-peer network of users who collectively adhere to agreed rules (which are insured by the software) for accepting new blocks. Each record in the block contains a timestamp or signature and a link to a previous block in the chain. By design, blockchain is made to ensure immutability of the data. So once recorded, the data in any given block cannot be modified afterwards without the alteration of all subsequent blocks and the agreement of the members of the network. Because of its integrity and immutability, blockchain could be used as an open, distributed ledger and can record transactions between different parties or networked database systems in an efficient, verifiable and permanent manner. It is also flexible enough to allow adding arbitrary logic to process, validate and access the data, which is implemented via so called smart contracts (components of business logic shared and synchronized across all nodes). This makes blockchain very suitable for application in healthcare and other areas where data is very sensitive and strict regulations on how data can be used need to be imposed.

## DATA PRIVACY ISSUES AND REGULATORY BARRIERS

### Data privacy issues

While data could be said to be the lifeblood of the current digital society, many are yet fully to grasp the need for appropriate acquisition and processing of data [52, 53]. Among the key concerns in the generation and use of data are privacy issues. This is even more important in healthcare, where a high percentage of personal health data generated could be considered private. In order to ensure propriety in the handling of data, there have been regulations and rules that guide processes such as generation, use, transfer, access and exchange of data. Although privacy has been recognized as a fundamental human right by the United Nations in the Universal Declaration of Human Rights at the 1948 United Nations General Assembly, there is yet to be universal agreement on what constitutes privacy [54]. As a result, privacy issues and regulatory concerns have often been topics of important but yet varied interpretations wherever data is generated and used.

### Regulatory barriers

With the dawn of computing and constant advancements in tech, there have been massive amounts of data generated on daily basis, and a substantial amount of these data consists of information which could be considered private. Some regulatory efforts to ensure proper flow and use of these data could become barriers to meaningful development [52]. Among the key efforts to ensure that data is used within the appropriate standards, is establishment of the Health Insurance Portability and Accountability Act (HIPAA) of 1996 and Privacy Rule’s minimum necessary standard [55]. While developers and researchers are usually keen to get down to work; analyzing, processing and using data, some barriers could make getting and using relevant data challenging [55-58]. While regulatory barriers like HIPAA are necessary to ensure appropriate use of information, they could delay developmental efforts, especially when meaningful work have to be done as fast as possible. For instance, HIPAA requires an institutional review board to approve the use of data, and this could simply introduce some degree of complexity to data use [57].

Most people believe that their medical and other health information is private and should be protected, and patients usually want to know how this information is being handled [59]. The transfer of medical records from paper to electronic formats could increase the chances of individuals accessing, using, or disclosing sensitive personal health data. Although healthcare providers and public health practitioners in the US traditionally protect individual privacy, previous legal protections at the federal, tribal, state, and local levels could be inconsistent and inadequate. Hence, the HIPAA was established to ensure health insurance coverage after leaving an employer, and also to provide standards for facilitating healthcare-related electronic transactions. With the aim of improving the effectiveness and efficiency of the healthcare system, HIPAA introduced administrative simplification provisions that required Department of Health and Human Services to adopt national standards for electronic healthcare transactions [60, 61]. Meanwhile, Congress realized that developments and advancements in computing and electronic technology could affect the privacy of health information. As a result, Congress added into HIPAA provisions that made the adoption of federal privacy protections for certain individually identifiable health information compulsory.

The HIPAA Privacy Rule (Standards for Privacy of Individually Identifiable Health Information) provides national standards for protecting the privacy of health information. Essentially, the Privacy Rule regulates how certain entities, also called covered entities, use and disclose individually identifiable health information, called protected health information (PHI). PHI is individually identifiable health information that is transmitted or maintained in any form or medium (e.g., electronic, paper, or oral), but excludes certain educational records and employment records [60, 62]. Among other provisions, the Privacy Rule:

1. gives patients more control over their health information;
2. sets boundaries on the use and release of health records;
3. establishes appropriate safeguards that the majority of health-care providers and others must achieve to protect the privacy of health information;
4. holds violators accountable with civil and criminal penalties that can be imposed if they violate patients’ privacy rights;
5. strikes a balance when public health responsibilities support disclosure of certain forms of data;
6. enables patients to make informed choices based on how individual health information may be used;
7. enables patients to find out how their information may be used and what disclosures of their information have been made;
8. generally limits release of information to the minimum reasonably needed for the purpose of the disclosure;
9. generally gives patients the right to obtain a copy of their own health records and request corrections; and
10. empowers individuals to control certain uses and disclosures of their health information.

It is absolutely important to maintain the privacy and security of health data, and Regulatory barriers serve to ensure rightful handling and use of these sensitive information. However, the complexity and difficulty introduced by these barriers could hamper meaningful progress in the use of data [57, 59]. There is therefore the need to develop systems and procedures that would not only ensure the appropriate handling and use of data but that would also significantly facilitate the use of data for meaningful progress towards better health outcomes.

## ADVANCES IN BLOCKCHAIN

The blockchain is a distributed database using state machine replication, with atomic changes to the database referred to as transactions grouped into blocks, with the integrity and tamper-resistance of the transaction log assured via hash links among blocks. The blockchain concept was introduced for Bitcoin in the context of decentralized electronic currency [63]. Blockchain is usually understood to be decentralized, jointly maintained by a plurality of independent parties (*maintainers*), with the security assumptions postulating that a certain fraction of these parties may be non-responsive or compromised at any moment during blockchain operation like Byzantine fault tolerance [64].

Here we briefly describe the key features of the public and private [65, 66] blockchain technology:

● Linked timestamping [67]: blockchain by design makes it possible to provide a universally verifiable proof of existence or absence of certain data or a state transition in the blockchain database. These proofs would be computationally unforgeable by third parties (i.e., anyone but a collusion of a supermajority of the blockchain maintainers), provided that underlying cryptographic primitives (hash functions and signature schemes) are computationally secure. Furthermore, accountability measures (e.g., proof of work or anchoring [68]) could make it prohibitively costly to forge such proofs for *anyone*, including the maintainers themselves, and provide long-term non-repudiation. Such proofs for small parts of stored data could be compact and do not need to reveal any other explicit information (only mathematically impersonal information).

● Blockchain uses a consensus algorithm [64, 69], which guarantees that non-compromised database copies have the same views as to the database state. In other words, consensus ensures that transactions in the log are eventually propagated to all non-compromised nodes and lead to the identical changes.

● Applied cryptography routines (e.g., public-key digital signatures [70]) are used to decentralize authentication and authorization of transactions taking place within the network. That is, transactions are created externally to the blockchain nodes, which limits the repercussions of a node compromise.

The blockchain users are commonly divided into three parts according to their roles:

● Maintainers of the blockchain infrastructure, who decide business logic on the blockchain. The maintainers store full replica of the entire blockchain data, thus have full read access to it and decide on the rules of transaction processing, and are active participants of the consensus algorithm on the blockchain, in other words, have write access to the blockchain. Importantly, the maintainers are bound with a formal or informal contract with the other users as to the business logic encoded in the blockchain. That is, the maintainers cannot set or change the transaction processing rules arbitrarily; indeed, they provide means for external users to audit the blockchain operation for correspondence to these rules.

● External auditors of the blockchain operation for example regulators, non-government organizations, law enforcement, who verify the correctness of the whole transaction processing in real time and/or retrospectively. Auditors are assumed to store replica of the entire blockchain data, or at least a logically complete portion of it, and read access to it to be able to perform complete audits. From the technical perspective, auditors do not participate actively in consensus, but otherwise are similar to maintainers in that they replicate the entire transaction log.

● Clients who are the end users of the services provided by maintainers. Each client may have access to a relatively small portion of blockchain data, but his/her software may utilize cryptographic proofs to verify, with reasonable accuracy, the authenticity of the blockchain data provided by maintainers and auditors.

For example, in Bitcoin, maintainers correspond to miners and mining pool software, auditors to non-mining full nodes, and clients correspond to simplified payment verification (SPV) wallets and, more generally, to client-side key management software. Generalizing Bitcoin network taxonomy, we will refer to nodes having read access to the entire blockchain as full nodes, which are subdivided into validator nodes and auditing nodes as per the roles described above; the software on the client side will be accordingly called client software.

By utilizing cryptographic accountability and auditability measures, blockchains could minimize trust and associated counterparty risk among participants in the system [71]:

● As transactions are cryptographically authorized by the logical originators of such transactions, blockchain eliminates the risks associated with the single point of failure posed by centralized authorization systems. Key management could be complemented with public key infrastructure that would tie authorization keys with real-world identities, if deemed necessary.

● Client-side data validation could allow reducing the risks associated with man-in-the-middle attacks, including those when MitM is performed on the server side (e.g., by compromising the user-facing backend of the system). The client-side validation could further utilize secure user interfaces and key management (e.g., TEE capabilities in modern mobile platforms).

● The universality of cryptographic proofs provided to clients could allow to reliably convey them to third parties (e.g., use electronic receipts provided by a blockchain managing supply chain, for tax accounting purposes or as evidence in legal action). Furthermore, cryptographic soundness of proofs allows to definitively restore the blockchain state even in the case when the maintainers are entirely compromised.

● The availability of real-time and retrospective authorization tools with guarantees of data authenticity could reduce costs of auditing and monitoring processes. This, in turn, could allow counterparties to more accurately assess the contract risks, and/or allow regulators to more precisely estimate systemic risks.

Blockchains could be categorized by the level of access to the blockchain data [66]:

● In public permissionless blockchains, all blockchain data is public. Furthermore, the consensus algorithm is censorship-resistant (e.g., proof of work used in Bitcoin), which ensures that maintainers are free to enter and leave the system; i.e., write access to the blockchain is public, too. The maintainers’ accountability in permissionless blockchains is achieved via economic means (e.g., prohibitively high cost of attacks in proof-of-work consensus).

● Private blockchains have a well-defined and restricted list of entities having read and write access to the blockchain (e.g., a group of banks, the regulator and law enforcement in a hypothetical banking blockchain). Notably, end users of services codified in the blockchain (i.e., bank clients in the example above) do not have any access to the blockchain data.

● Public permissioned blockchains restrict write access to the blockchain data similarly to private blockchains, but are engineered to be universally auditable and thus oriented for wide read access by end users. For the sake of brevity, in the following statement, we will refer to this kind of blockchains as permissioned.

## EXONUM FRAMEWORK FOR BLOCKCHAIN PROJECTS

Exonum (https://exonum.com, from Latin *exonumia*, numismatic items other than coins and paper money) is an open-source blockchain framework oriented towards permissioned blockchain applications with wide read access to blockchain data.

Exonum employs service-oriented architecture (SOA) [72] and architecturally consists of three parts: services, clients, and middleware.

● Services are the main extensibility point of the framework, which encapsulate business logic of blockchain applications. An Exonum-powered blockchain may have a plurality of services; the same service could be deployed on a plurality of blockchains (possibly with prior configuration). Services have a degree of autonomy in that each service is intended to implement logically complete and minimum necessary functionality for solving a particular task; their interface allows reuse and composability. In blockchain terms, services implement endpoints for processing *transactions* (cf. POST and PUT requests for HTTP REST services), as well as for *read requests* (cf. GET endpoints for HTTP REST services) that retrieve persistent information from the blockchain state (for the definition of blockchain state, see below).

● (Lightweight) clients implement typical functionality of clients in SOA; they are intended to be the originators of most transactions and read requests in the system, and are correspondingly supplied with cryptographic key management utilities, as well as tools to form transactions and verify (including cryptographically) responses to read requests.

● Middleware provides ordering and atomicity of transactions, interoperability among services and clients, replication of services among nodes in the network (which is purposed for both service fault-tolerance and auditability via auditing nodes), management of service lifecycle (e.g., service deployment), data persistence, access control, assistance with generating responses to read requests, etc. That is, middleware reduces the complexity of the system from the point of view of service developers.

The main advantages of Exonum for the described application compared to alternative permissioned frameworks are as follows:

● Because of design of data storage structures for auditability, Exonum could make it easier for clients and auditors (incl. ones with incomplete read access to data) to audit the system both in real time (incl. intermittently) and retrospectively. Further, the list of auditors could be unknown beforehand, and could be scaled over the course of blockchain operation.

● Due to use of service-oriented architecture, the application could easily reuse services developed for other Exonum applications, add and reconfigure services utilized for the application, etc. The service orientation and direct use of common transports (such as REST + JSON) could allow to streamline integration of third-party applications into the ecosystem provided by the Marketplace. Furthermore, service orientation could theoretically provide costless interoperability with other Exonum-based blockchains. (Albeit this possibility is not currently realized by the Exonum framework, the middleware layer could largely alleviate interoperability efforts needed to be pursued by service developers.)

● Compared to permissionless blockchains and frameworks with domain specific language/virtual machine indirection, Exonum provides substantially higher throughput capacity (order of 1,000 transactions per second), and ability to encode complex transactional logic, incl. interaction with external components.

● Exonum uses pessimistic security assumptions as to the validator node operation. The consensus algorithm employed in Exonum does not introduce single points of failure (e.g., dedicated orchestration / transaction ordering nodes). Furthermore, the set of validator nodes is reconfigurable, allowing to scale the security by adding new validators, rotating keys for validators, locking out compromised validators, etc.

### Blockchain storage

Blockchain state in Exonum is a persistent key-value storage (KVS), where keys and values are, in most general case, byte sequences of an arbitrary length, with the defined operations:

● Put a value under a specified key (creating the key if necessary)

● Remove a key-value pair by the key

● Iterate over keys in the lexicographic order, including starting from a specific key.

Exonum allows to split the key space of the common KVS into the hierarchy of typed collections: lists, sets and maps, whereas items of the collections (or key-value pairs in the case of maps) are binary-serializable as per the Exonum serialization format. Operations on these collections are mapped to the corresponding operations of the underlying KVS. The two uppermost levels of hierarchy correspond to services and data collections within a specific service, respectively; i.e., the 2^nd^ level of hierarchy are items of top-level service collections. Additional levels of hierarchy could be created by using collections as items of top-level collections.

Collections can be declared as *Merkelized*. Merkelized collections introduce a new operation, *hash* of the collection, which is the hash commitment to all its items (or key-value pairs in the case of map). This construction allows to create compact (logarithmic wrt the number of elements in the collection) cryptographic proofs of presence (and absence in the case of sets/maps) of items in the collection.

Similar to the hierarchical structure of collections within the blockchain described above, all Merkelized collections of a particular service could be committed to in a single hash digest (possibly, through one or more levels of indirection). Indeed, this hash digest could be calculated by creating a Merkelized meta-map of collection identifiers into collection hashes. Similarly, commitments of all services within the blockchain could be collected into a single blockchain-level hash digest, which would commit to *all* data in all Merkelized collections within the blockchain state. For all intents and purposes, the resulting blockchain-level hash digest is the hash (commitment) of the entire blockchain state. This would allow to create proofs of existence or absence tied to this single hash as a root of trust.

**Table 1:** Distinguishing characteristics of Exonum service endpoints

Characteristics	Transactions	Read requests
Localness	Global (subject to consensus)	Local
Processing	Asynchronous	Synchronous
Initiation	Client	Client
REST service analogy	POST / PUT HTTP requests	GET HTTP requests
Example on the cryptocurrency service	Cryptocurrency transfer	Balance retrieval

In order to reduce risks of history revisions and equivocation, H_state may be anchored on a permissionless blockchain with strong accountability guarantees (e.g., Bitcoin), and proofs provided to clients augmented accordingly. Cf. notion of partial proofs in the OpenTimestamps protocol (https://opentimestamps.org/). Note that the anchoring scheme would allow to reliably assert statements about the blockchain state retrospectively, even if the blockchain itself has become unavailable (e.g., due to wide-scale compromise or collusion of the blockchain validators).

### Network

Services may communicate with external world via 2 kinds of interactions:

● Transactions is the only way to change the blockchain state. Transactions are executed asynchronously, with their ordering and results of execution being subject of the consensus algorithm executed on the blockchain. For this reason, incoming transactions are broadcast among full nodes in the network

● Read requests allow to retrieve information from the blockchain state, which may be accompanied by the corresponding proofs of existence/absence. Read requests can be processed locally by any full node (or, more generally, by any node having sufficient read access to the relevant keyspaces of the blockchain state)

#### Transport layer

Due to universal verifiability of transactions and proofs, clients may connect to a single node for all requests. Note that maliciously acting node cannot forge proofs for read requests; but it could delay transaction processing by not broadcasting transactions received from the client. The transport protocol is not intended to be pinned by the Exonum specification; indeed, similarly to web services in frameworks such as Java EE and CORBA, the middleware layer is tasked with the responsibility to abstract transport layer functionality from service developers, so that invocation of service endpoints could be mapped to local method invocations. As of Exonum 0.2, RESTful JSON transport is supported for interaction of full Exonum nodes with clients, and TCP with a custom binary format is used in communication among full nodes.

#### Authentication and authorization

Transactions are necessarily authenticated by their originators with the help of public-key digital signatures to ensure their integrity, as well as real-time and retrospective universal verifiability. Public key infrastructure (PKI) could be built on top to achieve more complete non-repudiation and/or finely grained access control if necessary.

As read requests are local, authentication/authorization for them could be transport-specific, achieved, e.g., with web signatures (esp. for read requests implemented with the HTTP GET method) or by authenticating the communication channel (e.g., via client-authenticated TLS or Noise protocol).

In order to additionally boost security, service endpoints could be declared as *private*. Private endpoints could be compared with administrative interfaces in Web services; they are intended to process and manage local storage associated with a particular full node. Separation of private endpoints could simplify access control and decrease attack surface; e.g., if the HTTP transport is used, private endpoints are mapped to a separate listen address compared to other endpoints.

### Lightweight client

Most generally, a lightweight client in Exonum is a client-side library providing capabilities to communicate with full nodes (i.e., invoke service endpoints and receive responses) and cryptographically verify responses. A client could be complemented with key management capabilities and persistence of responses from full nodes; the former could be used for authentication of requests, and the latter could assist in non-repudiation and verifying consistency among different responses (e.g., monotonically non-decreasing blockchain height).

### Consensus

To order transactions in the transaction log and agree on the result of transaction execution, Exonum utilizes an authenticated, leader-based Byzantine fault-tolerant (BFT) [73] consensus algorithm. The Exonum network would continue operating even if up to 1/3 validators are hacked, compromised or switched off. Hence, there is no single point of failure in the network; the whole process of transaction processing is fully decentralized.

The consensus algorithm works under the assumption of unforgeable public-key digital signatures and a partially synchronous network. Under these conditions, the algorithm provides safety and liveness as defined in [74], with safety not depending on partial synchronicity. Similar to other partially synchronous BFT algorithms such as PBFT or Tendermint, the algorithm uses three kinds of consensus messages - block proposals, prevotes and pre-commits (see Consensus Section of the https://exonum.com/doc/ documentation), which are authenticated by digital signatures to enable transferring of messages among validators and to improve non-repudiation.

Compared to other leader-based BFT algorithms, the algorithm used in Exonum has the following distinguishing characteristics:

● Unbounded rounds: Voting rounds have a fixed start time, but do not have a definite end time. A round ends only when the next block is received or committed locally. This helps decrease delays when the network connection among validators is unstable.

● Work split: Block proposals include only transaction hashes; furthermore, transaction execution is delayed; transactions are applied only at the moment when a node receives enough prevotes for a proposal. Delayed transaction processing reduces the negative impact of malicious nodes on the system throughput and latency.

● Requests: Requests algorithm allows a validator to restore consensus-related information from other validators by utilizing the fact that all messages are digitally signed. This has a positive effect on system liveness.

The validator set is reconfigurable; validators could be added or removed by the agreement of the supermajority of existing validators. The same procedure could be used for key rotation for validators.

### Bitcoin anchoring

Exonum uses a BFT Bitcoin anchoring algorithm, which is packaged as a separate service. The algorithm periodically outputs the hash digest of a recent block on an Exonum blockchain, which commits to the entire blockchain state and transaction history, in a transaction on the Bitcoin blockchain. The anchoring transaction has a well-defined form and must be authenticated by a supermajority of validators on the anchored Exonum blockchain. Validators should use individual Bitcoin full nodes to get information from the Bitcoin blockchain in order to eliminate single points of failure associated with potential eclipse attacks [btc-eclipse] on the nodes. Anchoring transactions form a sequence; each next anchoring transaction spends an output created by the previous one. Authentication and chaining of anchoring transactions make the described anchoring procedure similar in its security characteristics to the paper-based anchoring described in [75].

## COMBINATION AND TIME VALUE OF DATA

Health care providers around the globe are tracking patient encounters through an electronic medical records system, generating terabytes of patient medical records. This giant amount of medical data is a gold mine of health information.

Each type of data (basic blood test, basic urine test, MRI, electroencephalogram, electrocardiogram, genome, transcriptome, microbiome etc.) and their combinations have relevant value, depending on quality of the medical records and its biological significance for certain disease condition (Figure 2). Different types of medical data have their own predictive value, representative sensitivity, prediction rate and weight. Patterns reflecting the changes in patient condition are more readable when doctor operates complex information, presenting patient health state on different levels at the current period of time. Some of the data types, like pictures, videos, voice can also have substantial predictive value for medical condition. For examples, several research groups already studied the application of voice and speech pattern recognition for diagnosis of Parkinson’s disease and its severity prediction [76, 77]

**Figure 1:**
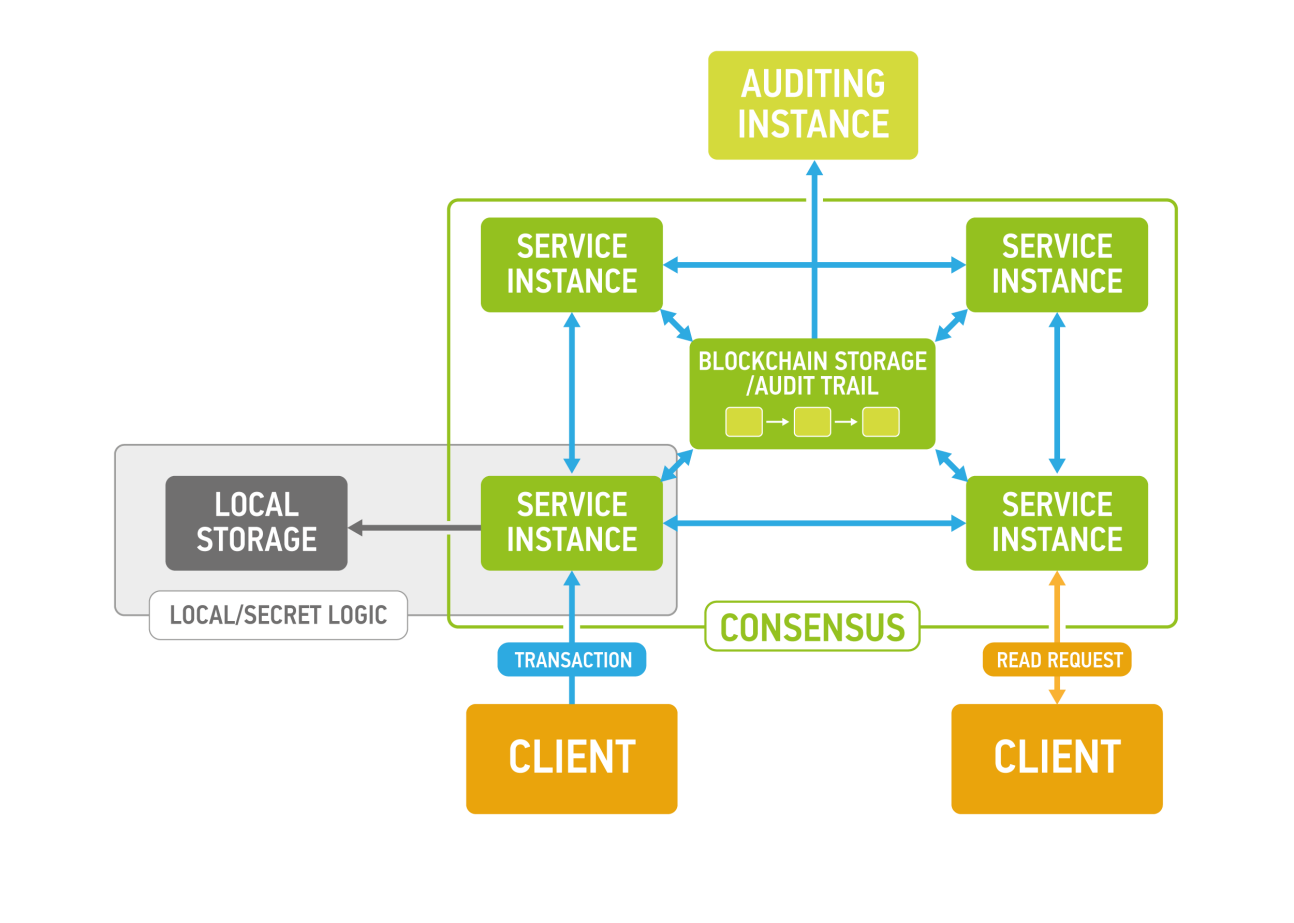
Exonum service design (each Service instance and Auditing instance has local replica of blockchain storage to ensure authenticity of the data and balance load)

**Figure 2:**
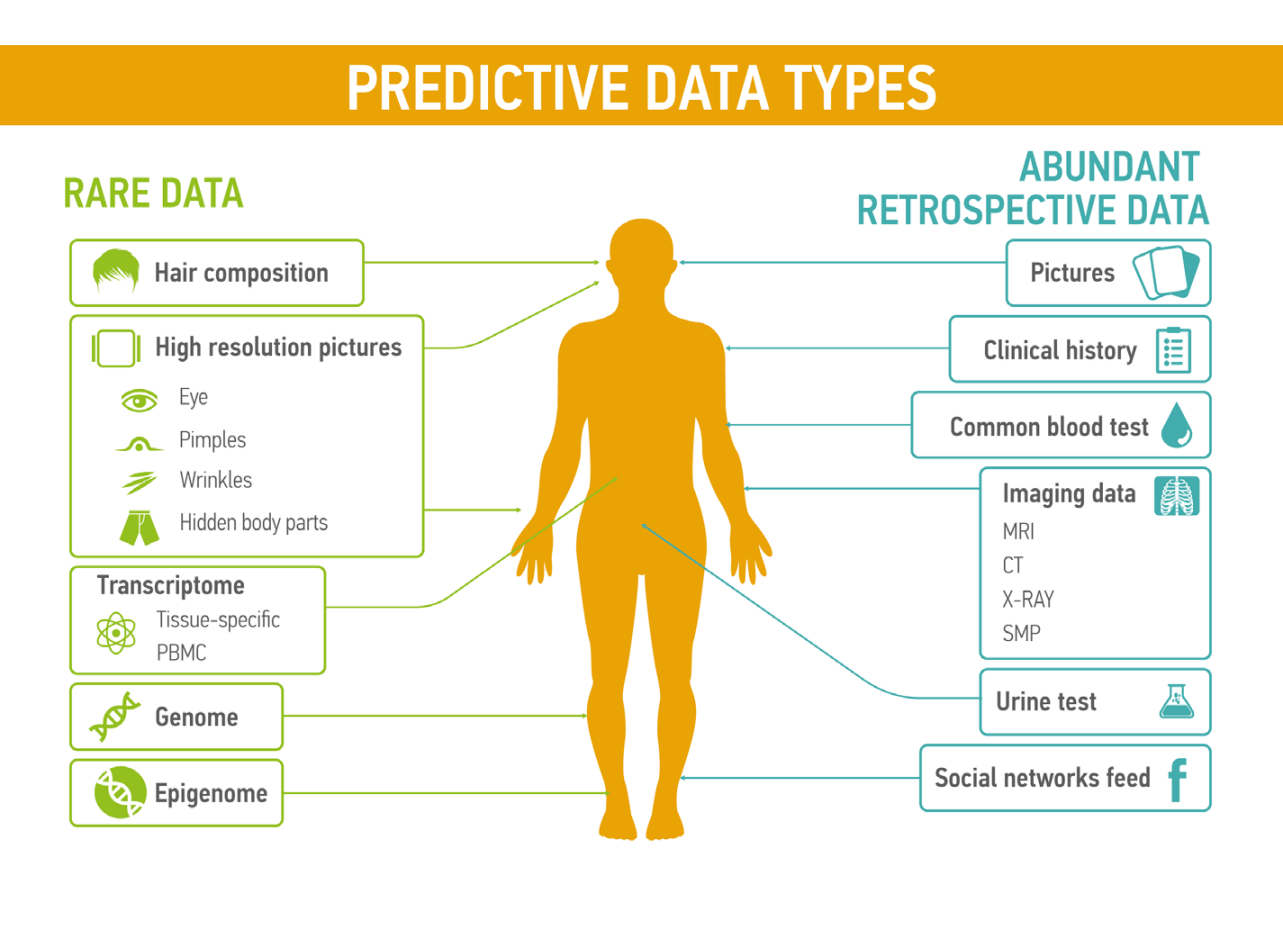
Predictive data types could be divided into two groups: rare data, such as the transcriptomic profiles, hair composition, or even novel data types that are not measured today and abundant retrospective data including common blood tests or the feed from social networks

Traditional diagnostics pipeline based on analysis combination of medical tests, especially when healthcare specialists try to diagnose serious and complex pathologies such as oncological, autoimmune, or neurodegenerative diseases. The combination of data types, especially the sum of low diagnostics data, provides a multi-level overview and better understanding of complex multifactorial conditions, and also leads to a faster diagnostics [78, 79]. Search and identification of suitable groups of biomarkers based on the multi-level data remains an important challenge. Taking into account different mechanisms of the disease development, biomarkers can acquire various forms. It is a common trend that various types of medical tests are being used for substantially broader diagnostic applications than those that were available at the start of their implementation.

Despite a large number of various diagnostic tests not all type of medical data is reasonable to use for the description of patient’s health state in dynamics. For example, genome analysis provides an important information on heredity, but due to it relative stability has a low value for prediction of dynamic changes in the organism compared to epigenome [80] or transcriptome [81]. Sampling time is an important component of any medical analysis, which allows to accurately describe the state of the human health at the moment. Following the principles of 360° health introduced by the NHS [82], the more different parameters are analyzed at the same time, the more detailed and voluminous the overall picture is. One-time combination of date provides a very nutritious feed for artificial intelligence, allowing to create powerful algorithms of effectively and precisely detection different human health states.

In this paper we introduce a formal model that allows to evaluate data value that takes into account combination and time parameters of data. Based on the value model one may establish a proper *cost* for using the given data combination, and this in turn allows for creation of formal medical data transaction model to create medical data marketplace.

### Data value model

Generally, data can be divided into the following categories: dynamic - reflecting the state of the organism at the time of sampling (blood test, transcriptome, epigenome, proteome, microbiome etc), and static - almost unchanged during the life of the patient (genome, fingerprint). Within a dynamic group, it is possible to differentiate rapidly changing data and gradually changing data.

In congenital genetic diseases, the records obtained in the first years of life are important as determining the further development of the disease (Figure 3.1), for the age-associated diseases, it is important to analyze the results obtained before the diagnosis was made (Figure 3.2), data role constant throughout the life of the patient (Figure 3.3).

**Figure 3:**
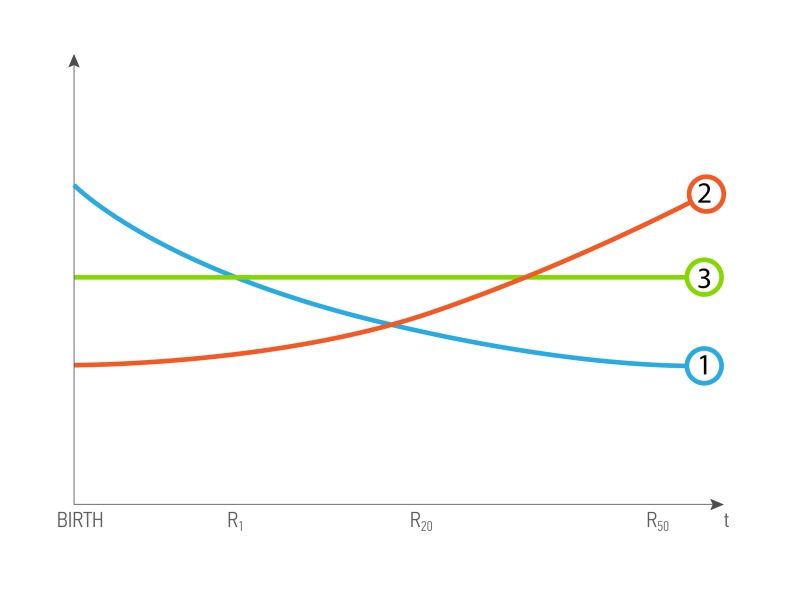
A possible scenario of data value dependence of age and health status of a patient R- biomedical record, and index is patient’s age. 1 - The curve of dependence for the R, when value decreases with time, and is most valuable in the young age 2 - The curve of dependence for the R, when value increases with time, and is most valuable in the old age. 3 - The curve of dependence for the R, when value is constant

Each personal biomedical record *R* could be viewed as a triplet (*type, time, quality*) where *type* is a categorical variable for a record type, *time* is a sampling time of patient’s biomedical record (for example, when blood test was made) minus the patient’s time of birth, *quality* is a nonnegative number reflecting record quality (generally, it could be a vector). One of the key attribute encapsulated in the *type* is the *half-life period* of analysis - characterizing the ½ duration of the relevance of the data. For example, according to one of largest medical practice and research center, Mayo Clinic, cholesterol check is valid for only five years or less if a patient at the higher risk of heart disease [83]. While, the genome profile is valid for the whole life of the patient, so genome analysis has longer *half-life period* than basic cholesterol blood test.

The dataset is a setDataset = {(user*r*_n_, *R*_n_)}_n=1_ of *N* (user*r*_n_, *R*_n_) pairs, where *user* is a user profile. User profile *(Patient’s profile)* - an attribute that includes information of ethnicity, date of birth, sex, diagnoses, blood type, medical prescriptions, vaccinations, chronic diseases, interventions, smoking and alcohol status, family relations, weight, height, geolocation. User profile refers to a hybrid attribute, since it includes both static (date of birth, ethnicity, sex, blood type) and dynamic parameters (diagnoses, smoking and alcohol status, weight, geolocation).

The dataset *Cost* is a function of a *Dataset* and it consists of two terms: the combination for a each single user and combinations for a set of same type records for a groups of users.

### Cost for single user

$$\mathrm{Cost}\left(\mathrm{user}\right)=\sum_{k=1\{\left\{i_{l},\ldots ,i_{k}\right\}|l<m:i_{l}<i_{m}}^{\infty }\sum_{\mathrm{AND}\left(\mathrm{user,}R_{i_{m}}\right)\in \mathrm{Dataset}\}}f_{k}\left(R_{i_{l}},\ldots ,R_{i_{k}}|\mathrm{user}\right).$$

where *k* is a number of records in a combination, all records in the combination are for the *user* and are different, *f*_k_ is a cost function for a combination for *k* records.

k=1:R=(type, time, quality)

$$f_{1}\left(R|\mathrm{user}\right)=\Psi \left(\mathrm{type}|\mathrm{user}\right),\times \mathrm{quality}\times \Psi \left(\mathrm{type}|\mathrm{user}\right)$$

where

*Ψ* (*type|user*) is a base value of given record type and user combintation. In the model we set it as a mapping of categorical parameter *type* to the positive numbers (*0, ∞*)

*Ψ* (*type|user*) is a time value of record. It is a function (*0, ∞*) → (*0, ∞*)

$$k>1:R_{1}=\left({\mathrm{type}}_{1},\;{\mathrm{time}}_{1},\;{\mathrm{quality}}_{1}\right),\ldots R_{K}=\left({\mathrm{type}}_{K},\;{\mathrm{time}}_{K},\;{\mathrm{quality}}_{K}\right)$$

In case of combination of several records we keep the cost component structure similar to *k* = 1 case. This leads to the need to define base value, quality and time value for interaction component to the cost of several records.

$$f_{k}\left(R_{1},\ldots ,R_{k}|\mathrm{user}\right)=\Psi_{k}\left({\mathrm{type}}_{1},\ldots {\mathrm{,type}}_{k}|\mathrm{user}\right)\times v_{k}\left({\mathrm{quality}}_{1},\ldots {\mathrm{quality}}_{k}\right),\times \;\Psi_{k}\left({\mathrm{time}}_{1},\ldots ,{\mathrm{time}}_{k},{\mathrm{type}}_{1},\ldots {\mathrm{,type}}_{k}|\mathrm{user}\right)$$

where

*Ψ*_*k*_ (*type*_*1*_*,....,type*_*k*_*|user*) is a base value of addition due to interactions. It is a mapping of categorical parameters *type*_*1*_*,....,type*_*k*_ to the positive numbers (*0, ∞*)

*v*_*k*_(*quality*_*1*_, . . . *quality*_*k*_) is a quality of combination of records for one user. It is a function [*0, ∞*)^*k*^ → [*0, ∞*) such that *v*_*k*_ is monotonic nondecreasing function of each input and _*quality*_lim_*m → 0*_ *v*_*k*_(*quality*_*1*_, . . . *quality*_*k*_) = 0 for all *m* = 1,...*k*. The last property means that the adding a record *R* with zero *quality* does not change the cost of the *Dataset*. For example, *v*_*k*_ = (Σ^*k*^_*m=1*_ 1/ *quality*_*m*_)^*-1*^

$$\Psi_{k}\left({\mathrm{time}}_{1},\ldots ,{\mathrm{time}}_{k},{\mathrm{type}}_{1},\ldots ,\;{\mathrm{type}}_{k}|\mathrm{user}\right)$$

is a time value for a set of records. For a fixed set of *time*_*1*_, . . .,*time*_*k*_ it is a function [*0, ∞*)^*k*^ → [*0, ∞*). For example, for each *type*_*m*_ two time parameters *T*_*m*_, *type*_*m,o*_ and nonnegative function *w*_*m*_ (*t*),*t* ≥ 0 is chosen. And

$$\Psi_{k}\left({\mathrm{time}}_{1},\ldots ,{\mathrm{time}}_{k},{\mathrm{type}}_{1},\ldots {\mathrm{,type}}_{k}|\mathrm{user}\right)=\max_{t}\;\min_{m}=1_{,\ldots k}w_{m}\left(\frac{t-{\mathrm{time}}_{\mathrm{m,}0}}{T_{m}}\right)$$

**Figure 4:**
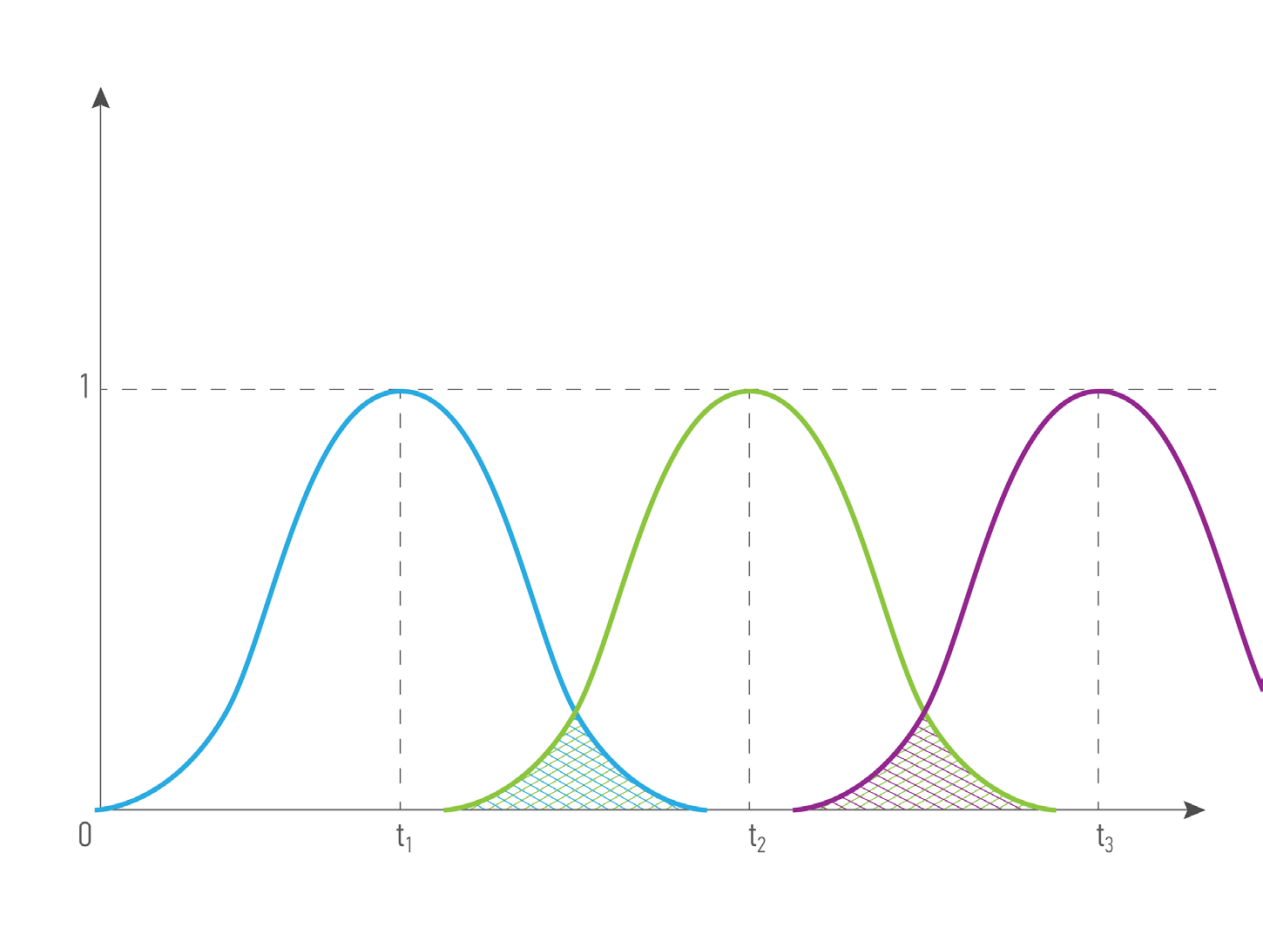
The cost of a combination of data (R_1_, R_2_, R_3_ ) of the same type obtained in different periods of time from the single patient, where *type***1** *= type***2***= type***3,** *quality***1** *= quality***2***= quality***3,** *time***1 ≠** *time***2 ≠** *time*_**3**_

**Figure 5:**
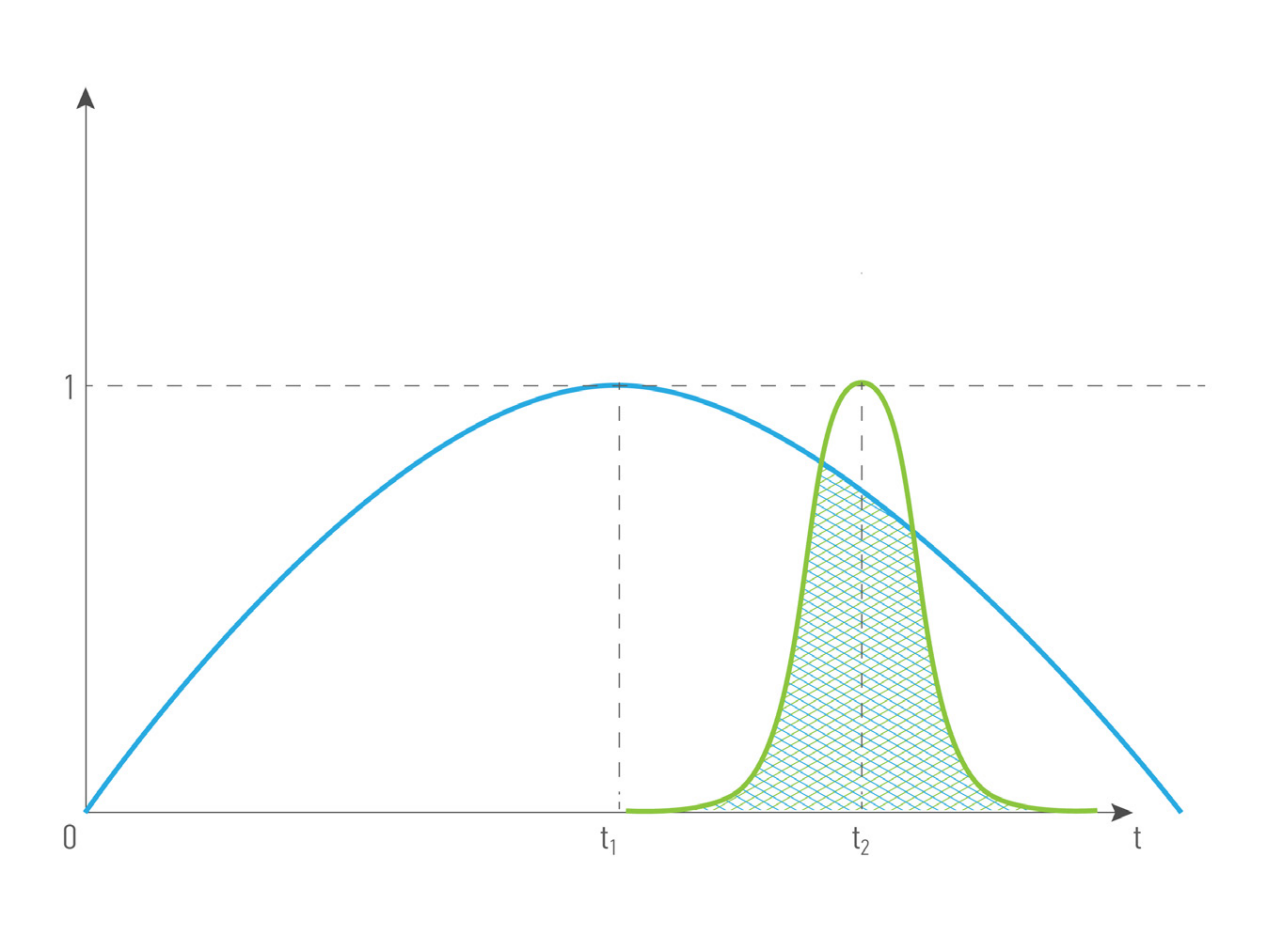
The cost of a combination of different data types (R_1_, R_2_ ) obtained in different periods of time from the single patient, where *type***1 ≠** *type***2,** *quality***1** *= quality***2***, time***1 ≠** *time***2** .

Figures 4 and 5 illustrate how the cost depending on records’ time were done: for the combination of the same type ( for example, blood tests made in different period of time) and for the combination of different types of data from the single patient (for example, blood test and transcriptome analysis). The greater intersection of time value curve is the greater combined records cost is. Data obtained it the same or short period of time have greater representation and predictive value, that’s why we introduce term *time value of data*. *Time value of data -* an indicator that demonstrates the representative and predictive rate of the group value of data, based on the difference in the records’ half-life time. It is relevant both: for a combination of data of one type and for a combination of data of different types.

### Cost for records from group of users

The cost increases only for the multiple records of the same type from distinct users. Let us fix *type* of records to find their combination cost increase. . Let *user*_i*1*_*,....,user*_i*k*_ have records with type *type* in the *Dataset* Let *quality*_i*1*_, . . . *quality*_i*k*_ be best corresponding qualities of users records with *type* in the *Dataset*. Then

$$\mathrm{Cost}\left(\mathrm{type},{\mathrm{quality}}_{i1},\ldots ,{\mathrm{quality}}_{\mathrm{ik}},{\mathrm{user}}_{i1},\ldots \;{\mathrm{user}}_{\mathrm{ik}}\right)=\gamma \left(Κ,\mathrm{type}|{\mathrm{user}}_{i1},\ldots ,{\mathrm{user}}_{\mathrm{ik}}\right)\times \frac{1}{K}\sum_{k=1}^{K}{\mathrm{quality}}_{\mathrm{ik}},$$

where for a fixed *type* function *γ*(*k*, *type|user*_*i1*_*,...., user*_*ik*_) has a fixed superlinear growth with *k* increase. For example, *γ*(*k*, *type|user*_*i1*_*,...., user*_*ik*_) = *C × K × lnk* or *γ*(*k*, *type|user*_*i1*_*,...., user*_*ik*_) = *C × K*^*3/2*^

Every Dataset has it’s own critical representative level, critical level depends on the type of data, their quality, and patient profile.

### Cost of buying dataset

If customer wants to buy *Dataset* and already has bought some *Dataset*^*1*^, then

$$\text{Cost}\left(\mathrm{Dataset}\right)=\text{Cost}\left(\mathrm{Dataset}\;U\;{\mathrm{Dataset}}^{1}\right)-\text{Cost}\left({\mathrm{Dataset}}^{1}\right)$$

The payments for users data are also fairly distributed among users according to their contribution to the dataset cost and previous payments from the current customer. Thus, the application of the data value model makes it possible to convert *Big Data* into *Apprised Data.*

### Family and relationship value of data

Many studies in healthcare require the data coming from the closely related subject from the same family or region and commonly involve Human data analysis is complicated redistricted experimental possibilities. However, these challenges could be overcome with a powerful and efficient design of data analysis. One of possible approaches is analysis of genetically close patients, twins, siblings or parents and offspring or colleagues and friends. , where observed effects are influenced by less number of features. Eventually,The biomedical data obtained for relatives is commonly more valuable than the same data obtained from the unrelated individuals.

Here we introduce a measure commonly used in the genealogy, the coefficient of relationship (*r*) between two individuals, also known as a coefficient of inbreeding, where a relationship between two subjects *B* and *C* is defined as

*r*_*BC*_ = Σ*p*_*AB*_*p*_*AC*_, where *p* is for path coefficients connecting *B* and *C* with common ancestor *A*.

andwhere *p*_*AB*_ is defined as:

$p_{\mathrm{AB}}=2^{-n}\times \sqrt{\left(\frac{\left(1+f_{A}\right)}{\left(1+f_{B}\right)}\right)}$, where *f*_*A*_ and *f*_*B*_ are inbreeding coefficient for ancestor A and offspring B, respectively.

Given the fact, that humans population are genetically heterogeneous and usually , or random-bred, we could set the*f*_*A*_=0 and a formula coefficient of relationship could be simplified:

*r*_*BC*_ = Σ*p 2*^*-L(p)*^, where *L(p)* is the length of the path *p*.

This way, *r* of parent-offspring is 2^-1^ = 0.5, , and *r* of grandparent-grandchild is 2^-2^ = 0.25.

And cost function of data could be modified as following:

$$f_{k}\left(R_{1},\ldots ,R|\mathrm{user}\right)=\Psi_{k}\left({\mathrm{type}}_{1},\ldots {\mathrm{,type}}_{k}|\mathrm{user}\right)\times v_{k}\left({\mathrm{quality}}_{1},\ldots {\mathrm{quality}}_{k}\right),\times \Psi_{k}\left({\mathrm{time}}_{1},\ldots ,{\mathrm{time}}_{k},{\mathrm{type}}_{1},\ldots {\mathrm{,type}}_{k}|\mathrm{user}\right)+\lambda \left[\Psi_{k}\left({\mathrm{type}}_{1},\ldots {\mathrm{,type}}_{k}|\mathrm{user}\right)\times v_{k}\left({\mathrm{quality}}_{1},\ldots {\mathrm{quality}}_{k}\right),\times \;\Psi_{k}\left({\mathrm{time}}_{1},\ldots ,{\mathrm{time}}_{k},{\mathrm{type}}_{1},\ldots {\mathrm{,type}}_{k}|\mathrm{user}\right)\right],$$

where λ is a regularization coefficient equals to coefficient of relationship of users the system and could be set as following :

λ Σ^*m*^_*i=1*_ *r*_*i*_, where *m* is a number of users in the system and *r* is a coefficient of relationship between them.

For distant relatives, *r* → 0 and almost will not contribute to the cost function of data, however, for a very close relative such as twins, *r* is equal to 1 and at the beginning, it will double the cost of data. The cost of data will grow with a number of close relatives that are using the platform and contributing their data.

## PREDICTING PATIENT’S AGE TO EVALUATE THE PREDICTIVE VALUE OF DATA

Chronological age is a feature possessed by the every living organism and one of the most important factors affecting the morbidity and mortality in humans. The multitude of biomarkers linked to disease are strongly correlated with age. For instance, triglycerides, glycated hemoglobin (HbA1c), waist circumference, IL-6 increase with age, but other parameters like albumin, IGF and creatinine clearance go in an opposite direction [84, 85]. Many efforts have been made to integrate biomarkers in various health/risk indexes like Healthy Aging Index [86, 87], Framingham Risk Score [88, 89], Frailty index [90, 91], Physiologic Index of comorbidities [92]. Ultimately, age is the closest estimate of a health status of a person. Hence, combining various biomarkers and linking them to age will provide the basis for platform able to provide integrative analysis of health status, assess data quality and even identify fake data. In addition, treating aging as a disease to train the deep neural networks to capture the most important biological properties of the age-related changes that transpire during aging using the deep neural networks facilitates for transfer learning on individual diseases using a much smaller number of samples. First proposed by Zhavoronkov et al in 2015 [93], this technique can be used to reconstruct the data sets with the missing or incorrect features.

**Figure 6:**
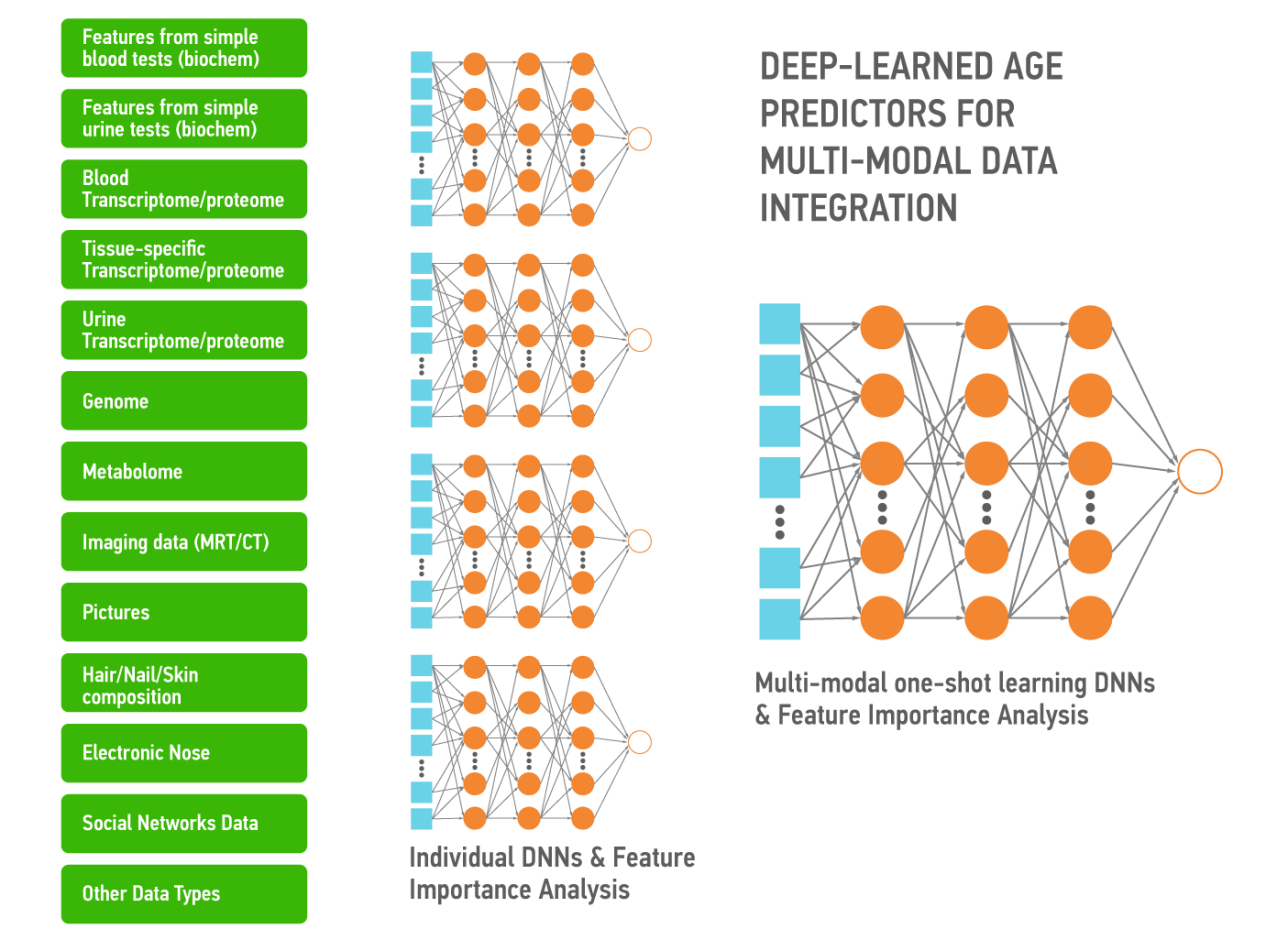
A simple depiction of the deep neural networks trained to predict the chronological age within the data type and using the features extracted using the feature importance and deep feature selection for multi-modal age predictors These predictors may be used for data integration, verification and transfer learning.

Aging is also a continuous process gradually leading to loss of function and the age-associated diseases. The DNNs trained on the multi-modal data ranging from photographs, videos, blood tests, “omics”, activity and even smell and sweat during aging capture the many biologically-relevant features about the group, individual, organ, tissue or even a set of molecules. These DNNs can be used to extract the features most implicated in aging and specific diseases to be used as targets or build association networks and causal graphs. These DNNs can also be re-trained on a much smaller number of data sets of specific diseases within the same data type or using the many types of biological data. Here we propose a high-level architecture fetaturing the various data types (Figure 6). First, for each data type we build a DNN predictor of chronological age for the reasonably healthy individuals. Individual DNNs will allow for the detection of outliers and data quality control. Then all individual DNNs will be used to train multi-modal one-shot learning DNN. This architecture allows not only for accurate age prediction, but also for feature importance analysis. Results of such analysis across all predictors will tell about the importance of each individual biomarker and may inform its relative ‘cost’. Since many of the biomarkers related to age (Albumin, Glucose, Norepinephrine, WBC, Il-6, etc.) are measured routinely in the clinic in a separate tests of different degree of invasiveness it is important to know which ones are more predictive.

## HEALTH DATA ON BLOCKCHAIN

One of the major problem for healthcare is data exchange and ability to use data in research and commercial projects. At the same time, healthcare sector requires to maintain a high standard of data privacy and security. Data breaches in healthcare storage systems can be especially costly because of HIPAA fines and reputation losses. Blockchain solutions as described later could reduce data breach risks by utilizing threshold encryption of data (meaning that cooperation of multiple parties is required to decrypt data), together with public key infrastructure (i.e., the use of asymmetric cryptography to authenticate communication with system participants).Gained a substantial attention in recent years T, the blockchain technology gained substantial popularity in recent years primarily due to the popularity of the Bitcoin crypto currency, was previously has been proposed as a medium for health care data storage solutions [94, 95] and as a tool for to improving thee transparency in clinical trials [96].

A blockchain-based system can dramatically simplify data acquisition process. They allow user to upload his data directly to the system and give his permission to use his data if it was bought through the system using transparent price formula determined by data value model. Also it would guarantee fair tracking of all data usage activity.

The promise of such solution is the opportunity for users to take ownership of their data and access priviledges and even allow them to sell their data directly to the consumers of data for the fair value of data. However, exchanging the data for currency may be problematic for many reasons including the need to perform a massive number of micro-transactions in multiple countries and among a large number of different types of the data market market participants.

Here we propose a new form of a utility crypto token called LifePound, which can be generated or mined by putting the data on the blockchain-enabled marketplace to facilitate for transactions and enable the novel incentive schemes.

**Figure 7:**
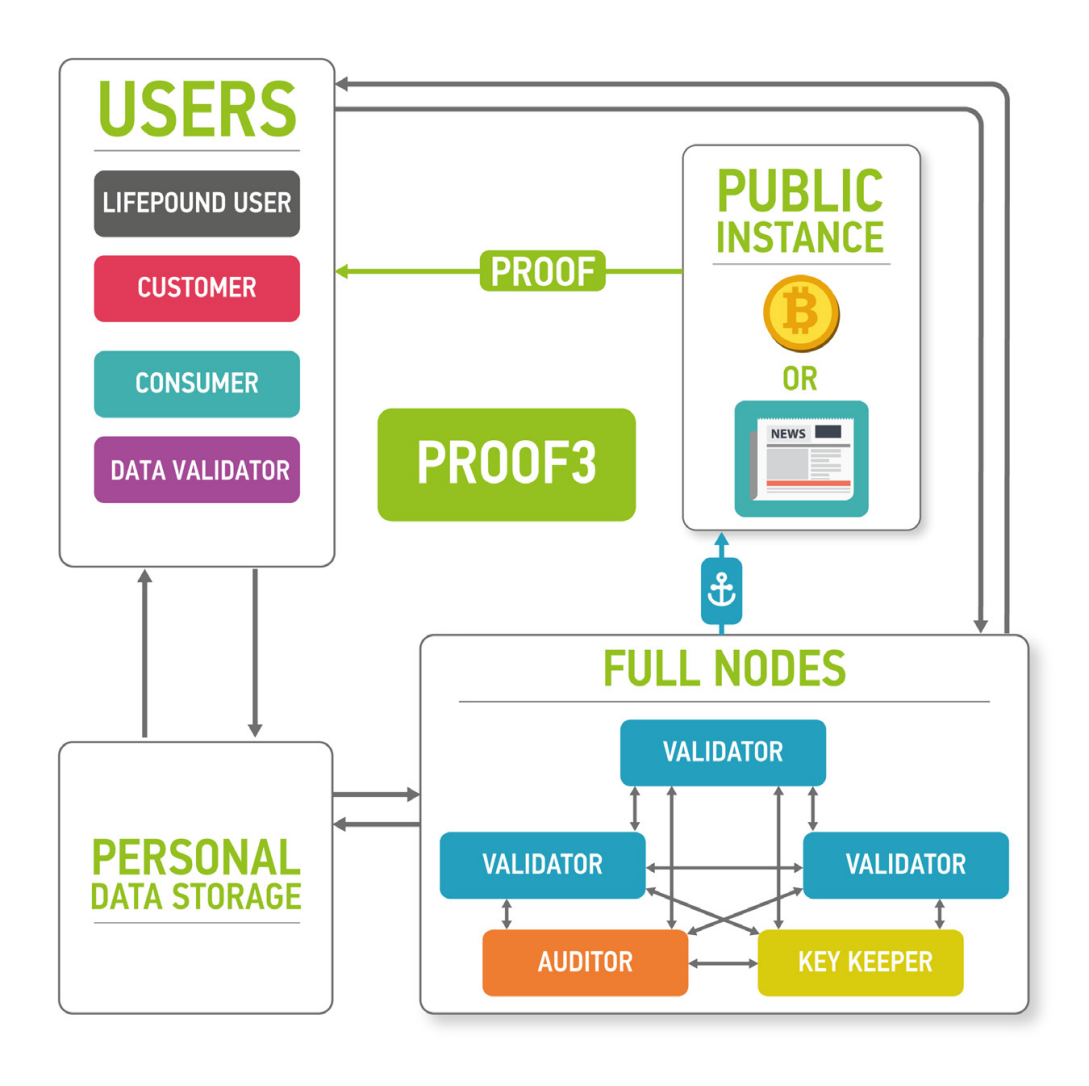
The marketplace ecosystem consisting of the four parts: blockchain part, data storage, users and public instances The blockchain is used to process new blocks of transactions, store and send keys and audit itself. The data storage contains encrypted data. Users send and sell their data using the marketplace (users), validate data (data validators), buy personal medical data (customers) and use LifePound as a cryptocurrency (LifePound users). The system is not fully open, and the public instances are used in cryptographic proofs for users to guarantee the marketplaces functioning correctness.

The architecture of the proposed platform is described in Figure 7.

The clients of the marketplace and their goals are

● users: to store and sell their biomedical data and to receive advanced health reports from the results of data analysis

● customers: to buy data from users and to provide results of data analysis for users

● data validators: to check the data received from users

● LifePound users: to use the cryptocurrency marketplace (possibly without any interaction with personal data).

Users are allowed to keep their data private and secured providing access to the data only for organizations whose paid for it and (optionally) staying as anonymous as possible. Customers intend to buy well-specified data samples which are aggregated from many users. To ensure the quality of the data provided by users, third party is needed - data validators, experts who are first buyers of the data. Data validators check the data quality and provide customers with a guarantee of user data validity. Interactions in the marketplace are registered on a blockchain in the form of transactions. Blockchain by itself does not contain any opened personal information. It contains hashes which could be used to timestamp and provide a reasonable level of non-repudiation for all actions at the marketplace. The former is achieved with the help of blockchain anchoring [52] and other accountable timestamping [59] techniques; the latter - with the help of digital signing and a blockchain-based PKI.

Blockchain full nodes and cloud storage are the remaining two parts of the ecosystem. Cloud storage could be an existing cloud storage, for example, Amazon Web Services (AWS), which allows for building HIPAA-compliant applications or Google Cloud Platform. One of the major reasons for integrating cloud storage into the ecosystem is to provide an off-chain storage solution especially for large biomedical data files, such as CT scans or MRIs, where the size of one data file could reach 50 Mb. The cloud storage may require authentication for read and write access to data, which in a preferable setup would be based on the PKI established on the marketplace blockchain. To ensure security and privacy, the data uploaded by users to the cloud storage would be encrypted on the user side using a threshold encryption scheme [97-100]. As the storage technology matures, it may be possible to replace cloud storage with the personal storage systems, where all the personal data would truly belong to the individual and also reside at the individual storage. The individuals also may be able to lend their data to the other parties for training purposes instead of selling the data.

Blockchain full nodes should be responsible organizations with an access to all information in the blockchain. They are divided into three subtypes:

● (Blockchain) validators: commit new blocks with transactions to the blockchain

● Auditors: audit the marketplace

● Key keepers: keep key shares according to a certain threshold encryption scheme necessary to decrypt user data in the storage. The precise protocol for key shares transmission and storage is out of the scope of this paper. In one possible setup, key keepers may have crypto-identities backed by a blockchain-based PKI, which would allow them to establish authenticated communication channels with other participants of the described protocol for key share transmission. In this setup, the keepers might use ordinary security mechanisms to guarantee at-rest security for the shares.

## CLIENT WORKFLOW EXAMPLES

The intersections between different marketplace participants are illustrated in this subsection using several client workflows.

## USER UPLOADS THE DATA

User chooses the data type and local path using system interface

1. User encrypts the data using a symmetric cipher (e.g., AES-256 in the CBC mode, or XSalsa20-Poly1305 authenticated encryption scheme used in libsodium [https://download.libsodium.org/doc/secret-key_cryptography/authenticated_encryption.html]). A Shamir’s secret sharing technique [101] is then used to split the secret key to be distributed among key keepers, so that any K of key keepers together would be able to decrypt data, where K is a constant less than the number of key keepers N. The choice of constant K depends on the blockchain security model; as per Byzantine fault tolerance assumptions, *K* > *round* (*N*/3).
2. User distributes key shares among key keepers, e.g., using a direct authenticated communication channel established with each keeper.
3. After user uploads encrypted data on a cloud it is consider to be LifeData.
4. User generates a transaction for data upload in order to notify ecosystem participants (in particular, data validators) that the upload has taken place. The transaction contains user’s public key, data type, and a link to the data at the cloud storage.
5. User signs the transaction and broadcasts it to blockchain nodes.
6. The transaction is included into the blockchain via consensus algorithm.
7. Now data validators can buy this data for validation.

## DATA VALIDATOR VALIDATES THE DATA

1. Data validator (DV) chooses the (batch of) unvalidated data and generates a transaction to buy it for the validation.
2. DV signs transaction and broadcasts it to the blockchain nodes.
3. The transaction is included into the blockchain via consensus algorithm. If the DV has enough LifePounds to buy it for validation, the DV’s LifePounds are sent to a validation smart contract and the workflow goes to step 4. Otherwise, the DV fails to validate data and goes to step 1.
4. Key keepers see an actionable data validation transaction in the blockchain. Each key keeper delivers stored key shares for each piece of data in the batch to the DV, e.g., via an authenticated communication channel.
5. DV uploads encrypted data from the cloud storage.
6. Once DV receives enough key shares from the key keepers, he decrypts the data.
7. DV validates data. The result is a vector of boolean values, signaling if corresponding pieces of data in the batch are valid or invalid w.r.t. the validation model used by the DV. The time for validation is limited. If validator failed to validate the data, the smart contract for data validation defaults to deeming all data in the batch valid.
8. DV forms and signs the transaction for data validation. The transaction contains the hashes of data and the validation result.

If the result for a particular data item in the batch is “valid”, then

● LifePounds from smart contract are distributed among the data submitters’ accounts according to the data value model described in the previous section

● Validated data becomes available for sale on the platform

DV will get a share of the revenue from third parties purchasing data from the batch in the future (see the following section). In one setup, the share of the revenue allocated to the DV is a blockchain-wide parameter.

If the result for a particular data item in the batch is “not valid”, then

● LifePounds from smart contract are refunded to the DV’s account

● Validated data is not available for sale at the platform.

*Note. It is reasonable to have several DVs*.

## CUSTOMER BUYS THE DATA

1. Customer chooses the (batch of) validated data and generates a transaction to buy it.
2. Customer authenticates the transaction and broadcasts it to the blockchain nodes.
3. The transaction is included into the blockchain via consensus algorithm. If the customer has enough LifePounds to buy the specified data, the workflow goes to step 4. Otherwise, the customer fails to buy data and goes to step 1.
4. Key keepers see an actionable data purchase transaction in the blockchain. Each key keeper sends the key shares for all data in the batch and securely transmit them to the customer (e.g., via an authenticated communication channel).
5. Customer downloads encrypted data from the cloud storage.
6. Once the customer receives enough key shares from key keepers, he decrypts the data.

## DATA SECURITY AND PRIVACY

One of the major challenges in the data-driven healthcare is data security and data privacy. Both the consumer, healthcare and research companies require the data of the many individuals to train their deep neural networks. The companies with the largest data sets acquire the data in the ways that may not be very transparent to the individuals and often the companies and the individuals do not understand the value of this data. The regulators often set up the barriers for the consumer data collection and storage substantially inhibiting the propagation of the recent advances in AI into the clinical practice (Figure 11).

**Figure 8:**
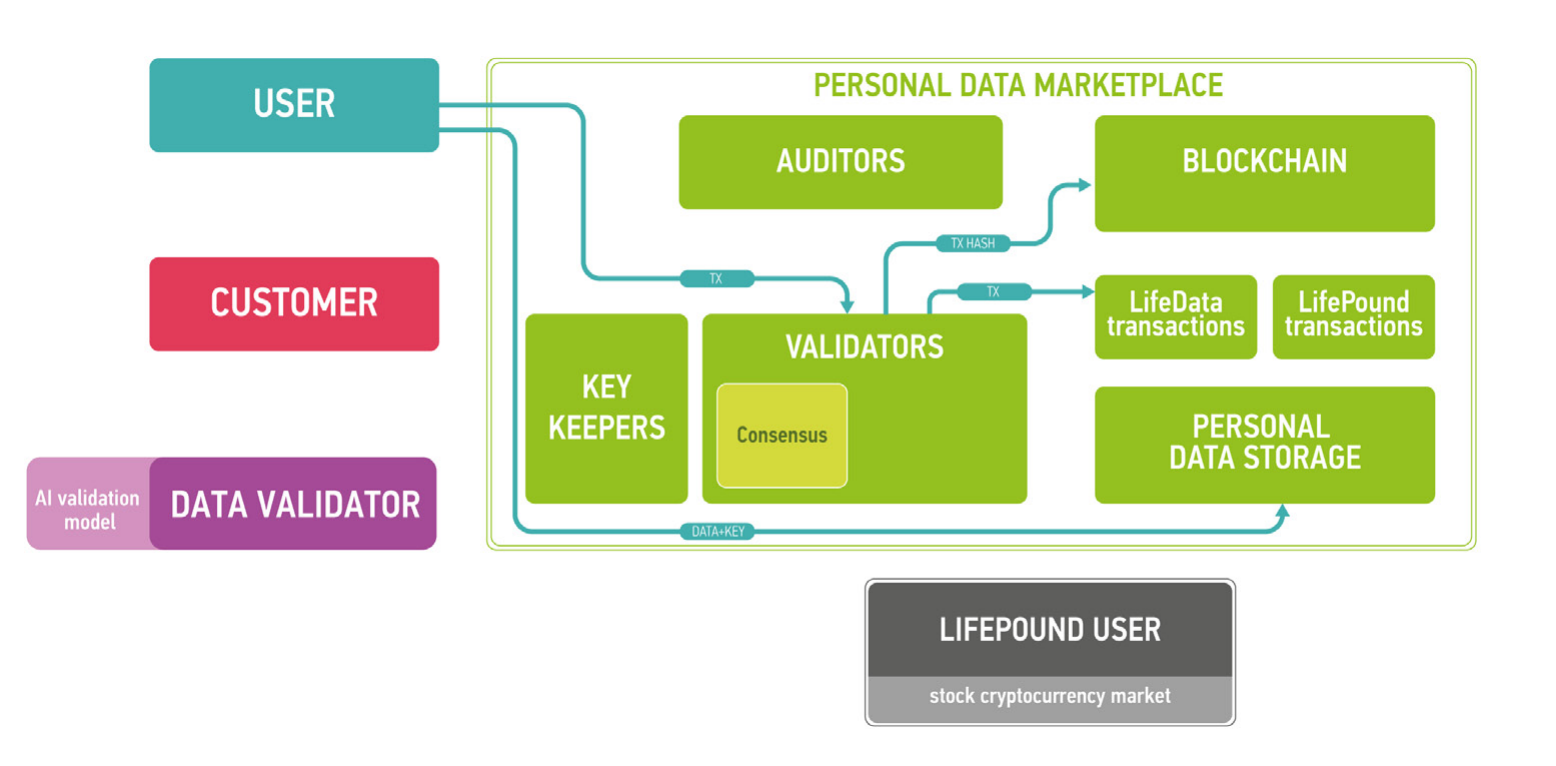
The workflow example for marketplace users User uploads data and gets LifePounds as a reward (amount depends on the value of data).

**Figure 9:**
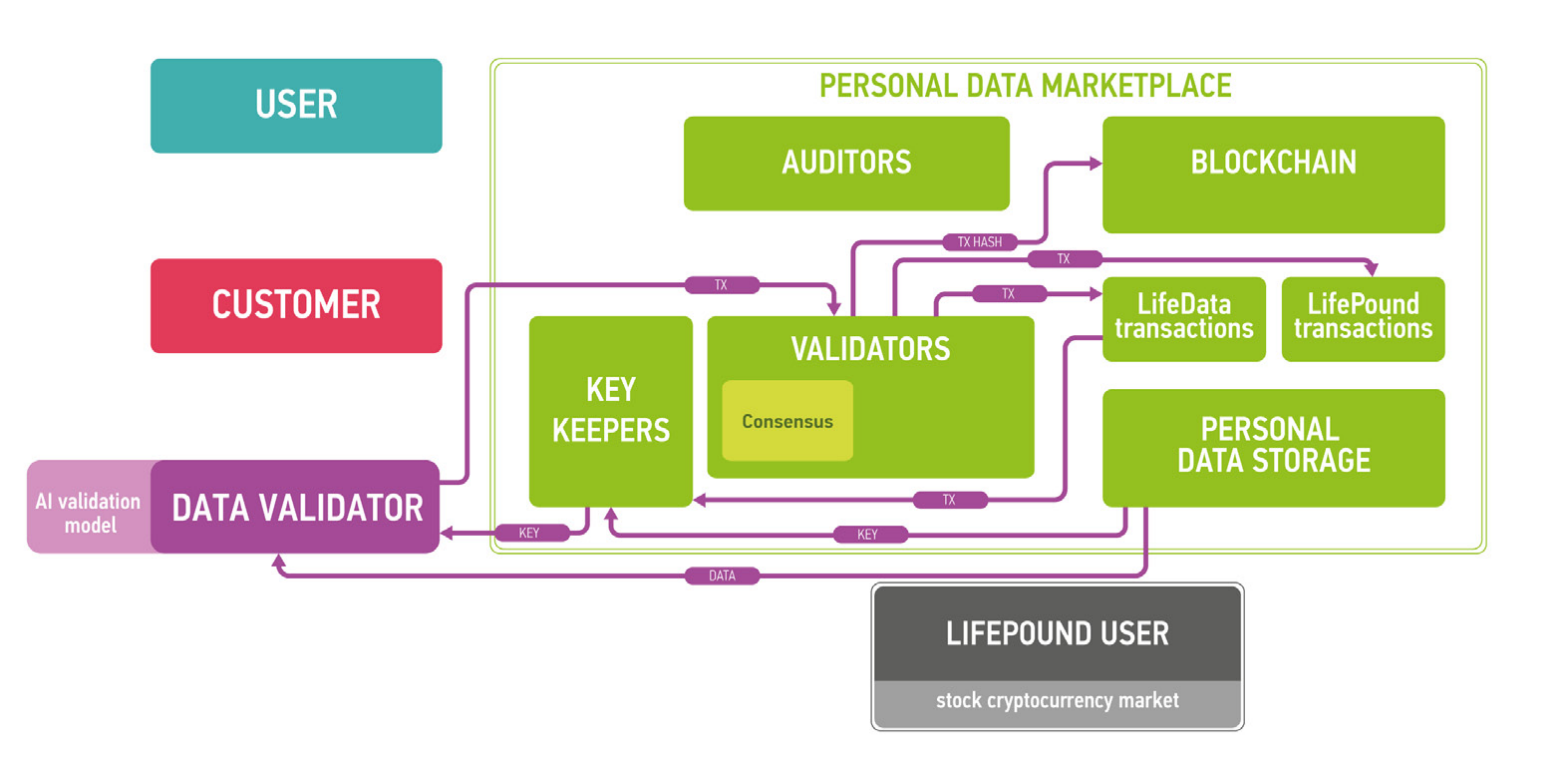
The workflow example for marketplace data validators (DV) Data validators are intermediate data buyers, who provide validation services to mine Lifepounds.

**Figure 10:**
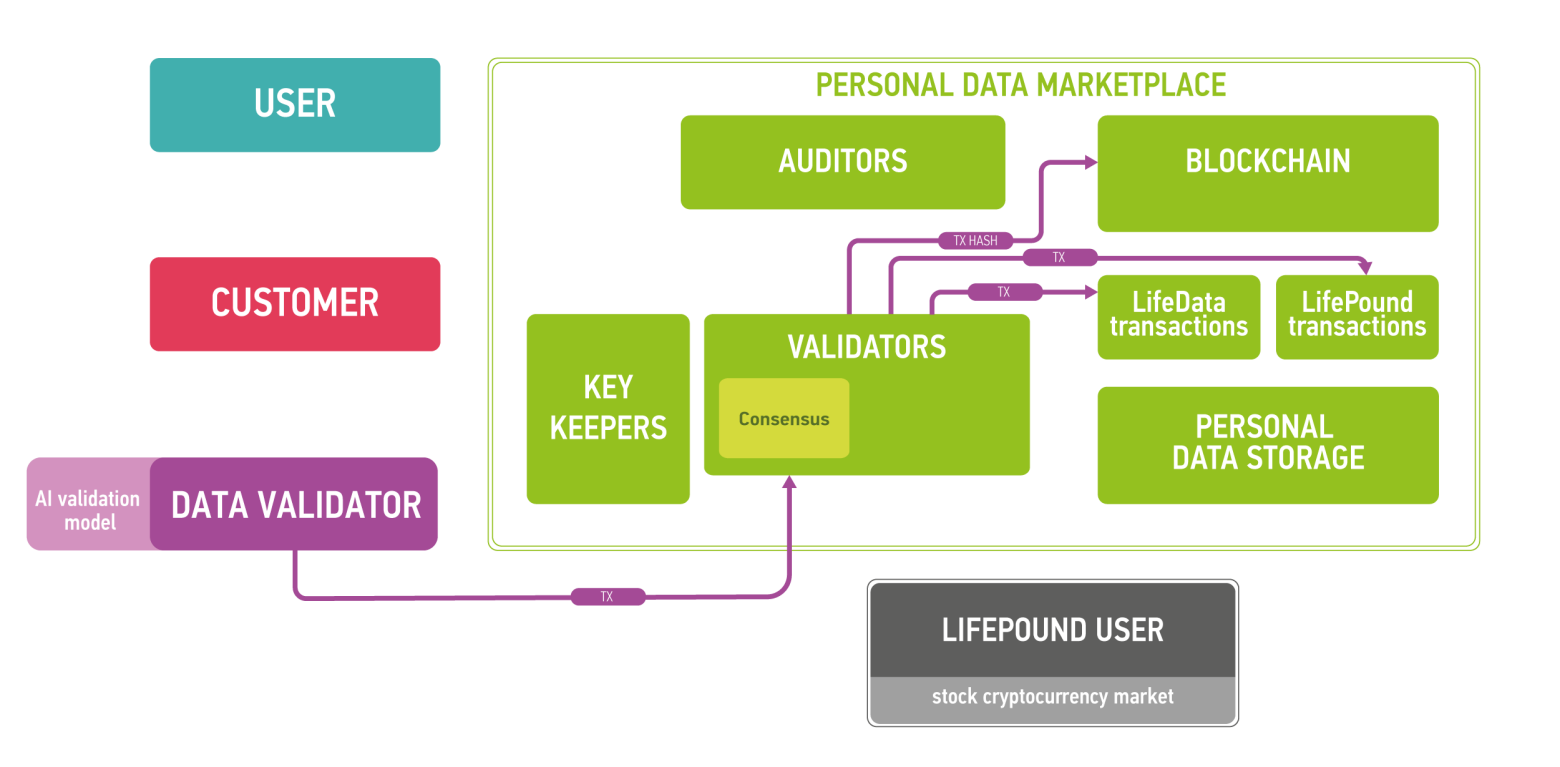
The workflow example for marketplace customers Customers buy data for LifePounds. The data value model determines the data cost.

**Figure 11:**
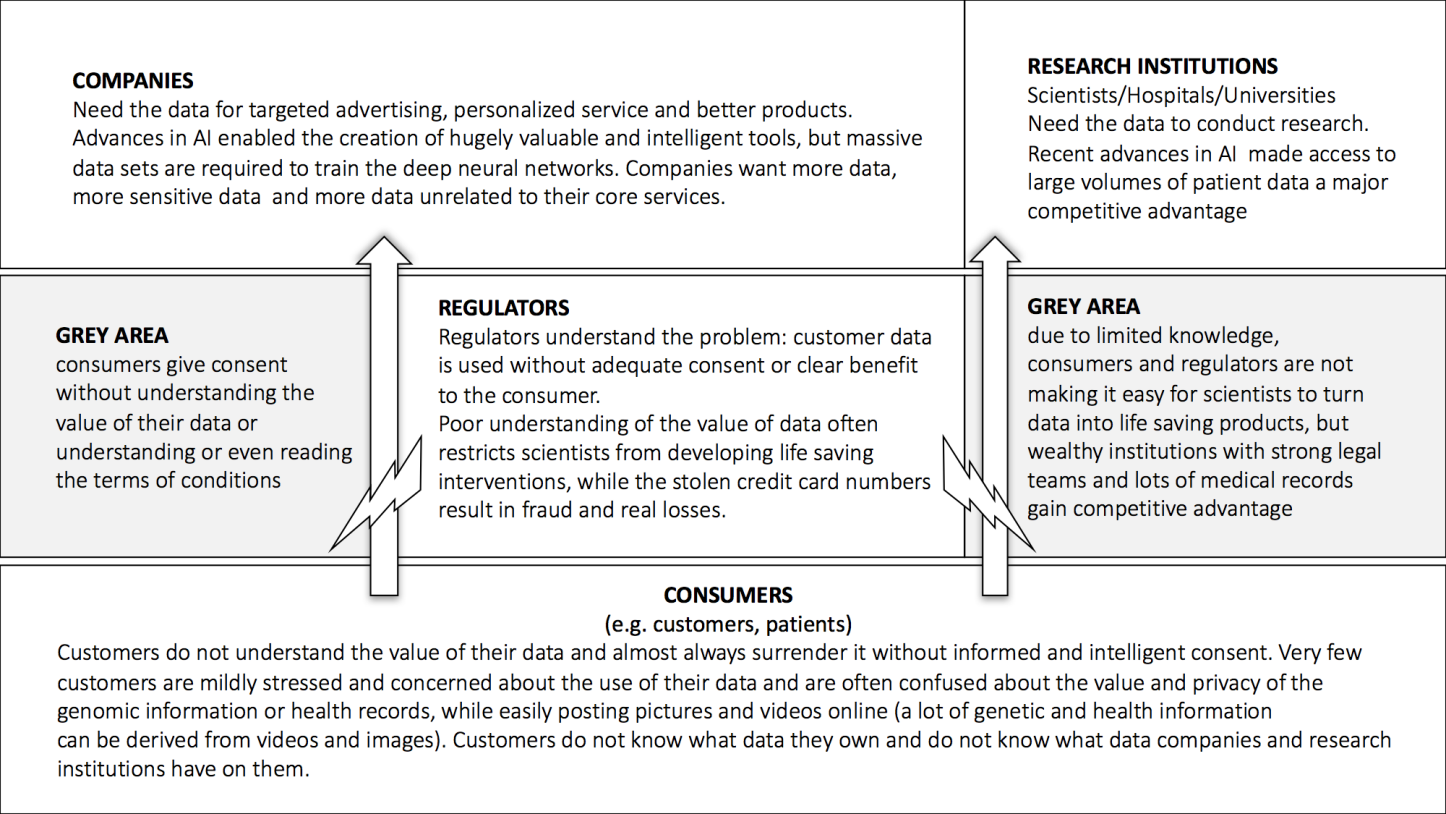
The flow of data from the individuals to the companies and research institutions The introduction of the blockchain-based data ecosystem may help ensure that the individuals take control over their data and companies and research institutions may acquire data more freely reducing the need for the regulators to interfere.

The security of the described setup relies on the security of utilized crypto-primitives: the hash function and public-key signature scheme(s) utilized in the marketplace blockchain construction, as well as the symmetric cipher(s) and the secret sharing scheme(s) used for encrypting user data. Compared to centralized setups, the proposed scheme could allow to alleviate several attack vectors:

● Blockchain-based PKI for logically “well-known” users (e.g., blockchain validators, data validators, key keepers, etc.) could base on well-established measures for secure key management (e.g., key sharing, use of specialized hardware for key storage, etc.). These measures could be augmented with blockchain-based smart contracting (e.g., multi-signatures); further, blockchain could provide secure facilities for monitoring key revocation and issuance, which remain the weakest points for centralized PKI setups.

● The use of threshold encryption could allow to alleviate a single point of failure in long-term data storage. As data in the storage would be encrypted, the compromise of the storage would not lead to the data leakage. (Note, however, that access to the storage should be additionally restricted, e.g., by authenticating storage users with the help of the PKI established on the marketplace blockchain.) The compromise of a single key keeper likewise would not lead to the data compromise, as its key shares would be insufficient to decrypt data in the storage.

● The use of authenticated communication channels to transmit key shares could allow to achieve forward secrecy, i.e., non-compromise of the encrypted user data even if the key keepers’ long-term asymmetric keys would become compromised (incl. the compromise of the underlying asymmetric cryptosystem, e.g., with the advent of quantum computing).

The user-side key management for data encryption and authentication of blockchain transactions may be prone to various risks (e.g., a faulty random number generator resulting in generation of keys with insufficient entropy; inadequate security for long-term key storage; compromises of the user interface). Minimizing these risks requires careful design of the client software and supporting materials. Existing solutions for cryptocurrencies and/or generic key management may be adapted to reduce the risks.

The described setup does not concern data safety (in particular, protection against leakage) after the data has been purchased and transferred to the buyer. Such protection could be achieved with the help of existing security measures for data at rest and in use, and therefore is out of the scope of the present paper.

## DEEP LEARNING FOR DATA QUALITY AND CONSISTENCY

While the DNNs are considered to have an exceptional generalization ability, they could be biased by the data they are trained on. Data quality is crucial for data-driven models; however, at the same time those models could be applied for data quality control and perhaps are the most suitable solutions for this task. First group of methods that could be utilized for healthcare quality check are unsupervised models aimed to detect anomalies that cluster far from the dataset. Deep autoencoders as unsupervised approaches which having as outputs input data itself and could be trained to reconstruct data also are suitable for anomaly detection. Poor-quality samples could be recognized as points with the highest reconstruction error [102]. Another set of approaches for the task are time-series based models, such as RNNs. Distribution of normal or good quality samples first is learned and then tested on a few next points in order to adjust model behaviour to the bias in the dataset not linked to the anomaly/poor quality samples. Anomalies in the data could also be linked to health conditions, so both those approaches could be used for pathology detection in health recordings [103, 104]. Finally, set of supervised techniques could be applied for data quality control [105]. However, one should take into account that supervised models require labelled dataset, which in case of anomaly detection will be highly unbalanced. Still, the problem could be solved with help of zero and one shot learning.

## CONCLUSIONS

In this paper we presented the first attempt to assess the value of time and the combination value of personal data in the context of an AI-mediated health data exchange on blockchain.The value of the various types of data, combinations of the various data types, time value of one data type and time value of combination of data types is poorly understood and often debated. To address this problem, we foresee the emergence of a new profession “data economist” and creation of the health data economics research institutes. Recent advances in artificial intelligence enabled the creation of highly accurate predictors of biologically relevant features such as age, race and sex from very simple data types such as selfies, blood tests and such. The value of the various data types may depend on the application. For example, for the insurance companies, while the cost of data generation may be significantly higher for the genome compared to a selfie, the value of the recent picture of the patient may significantly exceed the value of the genome, since it may be more predictive of the patient’s age, health status and mortality. However, the combination of these data types will be considerably more valuable than the value of these data types individually.

**Figure 12:**
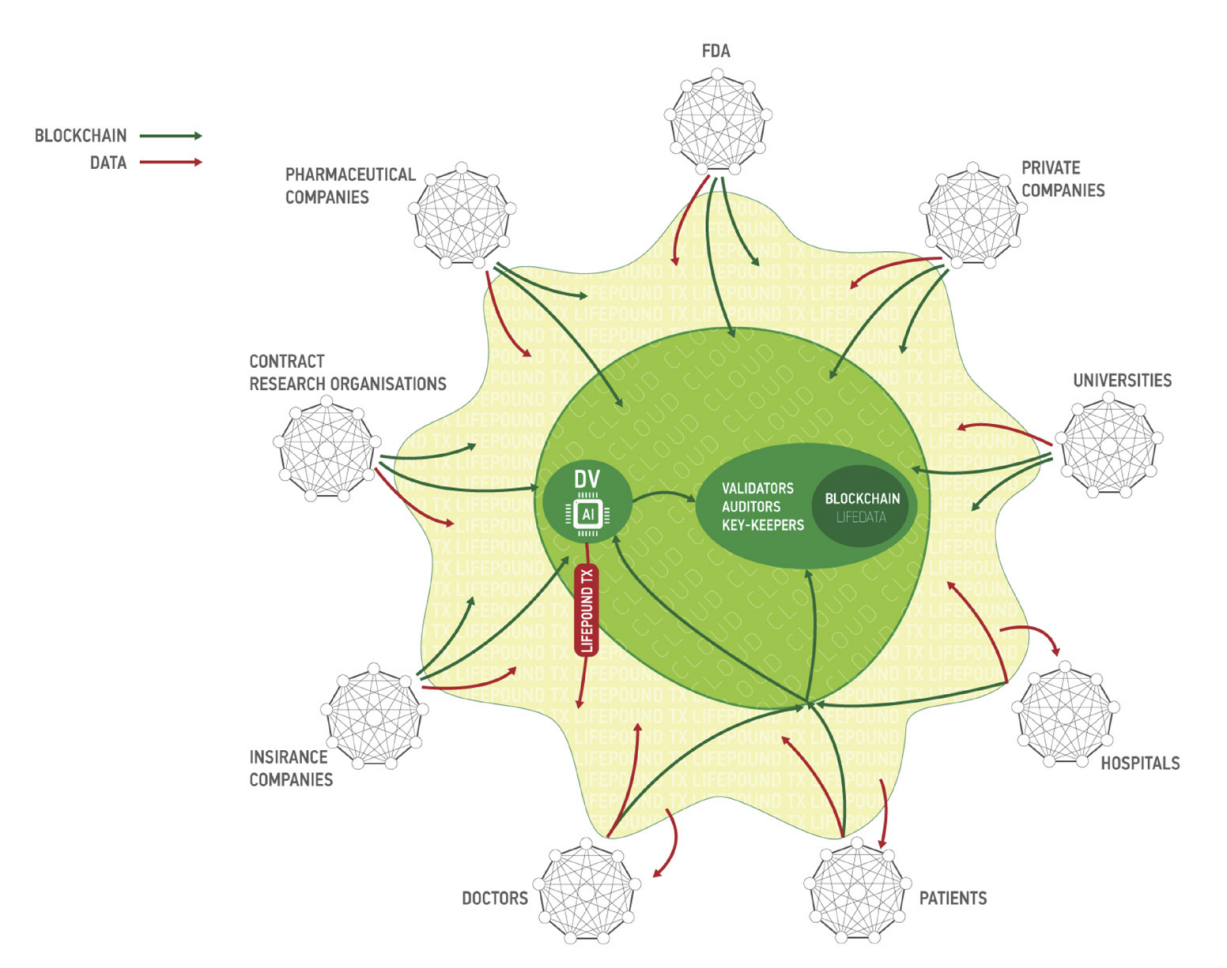
Proposed personal data-driven economy, where the individuals have full knowledge of and control over their data and are rewarded for generating new data and for providing the data for research or commercial purposes Such ecosystem may allow the regulators including the Food and Drug Administration (FDA) and the pharmaceutical and consumer companies to exchange their data

Blockchain and AI open new paradigms for health data ecosystems (Figure 12).

Blockchain technology enables the creation of a distributed and secure ledger of personal data, where patients are in control, own their data, and monitoring of access privileges and understanding of who looked at the data. Most importantly, blockchain technology allows for the creation of a data-driven marketplace, where patients can earn tangible rewards for making their data available to the application development community, pharmaceutical and consumer companies, and research institutions and generating new data through regular and comprehensive tests and checkups. Presently, only a few patients worldwide have the comprehensive data sets containing their clinical history combined with the genetic, blood biochemistry and cell count profiles, lifestyle data, drug and supplement use and other data types, because they do not see the value in this data and do not get tested regularly. On the other hand, the pharmaceutical and consumer companies alike are willing to pay substantial amounts for the large numbers of personal data records required to train their AI. These funds can be used to subsidize the regular testing by the patients, uncover the new uses for the various data types and develop sophisticated diagnostic and treatment tools.

## Abbreviations

NHS: National Health Service
DNN: Deep Neural Network
CNN: Convolutional Neural Network
RNN: Recurrent Neural Network
AUC: Area Under the Curve
LSTM: Long Short-Term Memory
MRI: Magnetic Resonance Image
GAN: Generative Adversarial Network
HDSS: Highly distributed storage systems
HIPAA: Health Insurance Portability and Accountability Act
PHI: protected health information
SPV: simplified payment verification
SOA: service-oriented architecture
KVS: key-value storage
PKI: Public key infrastructure
BFT: Byzantine fault-tolerant
